# Tumor cell integrin β4 and tumor stroma E-/P-selectin cooperatively regulate tumor growth in vivo

**DOI:** 10.1186/s13045-023-01413-9

**Published:** 2023-03-17

**Authors:** Sandra Genduso, Vera Freytag, Daniela Schetler, Lennart Kirchner, Alina Schiecke, Hanna Maar, Daniel Wicklein, Florian Gebauer, Katharina Bröker, Christine Stürken, Karin Milde-Langosch, Leticia Oliveira-Ferrer, Franz L. Ricklefs, Florian Ewald, Gerrit Wolters-Eisfeld, Kristoffer Riecken, Ludmilla Unrau, Linda Krause, Hanibal Bohnenberger, Anne Offermann, Sven Perner, Susanne Sebens, Katrin Lamszus, Linda Diehl, Stefan Linder, Manfred Jücker, Udo Schumacher, Tobias Lange

**Affiliations:** 1 Institute of Anatomy and Experimental Morphology, University Medical Center Hamburg-Eppendorf, Martinistrasse 52, 20246 Hamburg, Germany; 2 Institute of Anatomy I, Cancer Center Central Germany, Jena University Hospital, Teichgraben 7, 07743 Jena, Germany; 3 Department of Anatomy and Cell Biology, University of Marburg, Robert-Koch-Strasse 8, 35037 Marburg, Germany; 4 Department of General, Visceral and Thoracic Surgery, University Medical Center Hamburg-Eppendorf, Hamburg, Germany; 5 Department of General, Visceral and Thoracic Surgery, University Hospital Cologne, Kerpener Strasse 62, 50937 Cologne, Germany; 6 Faculty of Medicine, MSH Medical School Hamburg, Medical University, 20251 Hamburg, Germany; 7 Department of Gynecology, University Medical Center Hamburg-Eppendorf, Hamburg, Germany; 8 Department of Neurosurgery, University Medical Center Hamburg-Eppendorf, Hamburg, Germany; 9 Institute of Biochemistry and Signal Transduction, University Medical Center Hamburg-Eppendorf, Hamburg, Germany; 10 Department of Pediatric Hematology and Oncology, Research Institute Childrens’ Cancer Center, University Medical Center Hamburg-Eppendorf, Hamburg, Germany; 11 Research Department Cell and Gene Therapy, Department of Stem Cell Transplantation, University Medical Center Hamburg-Eppendorf, Hamburg, Germany; 12 Institue of Experimental Immunology and Hepatology, University Medical Center Hamburg-Eppendorf, Hamburg, Germany; 13 Institute of Medical Biometry and Epidemiology, University Medical Center Hamburg-Eppendorf, Hamburg, Germany; 14 Institute of Pathology, University Medical Center Göttingen, Robert-Koch-Strasse 40, 37075 Göttingen, Germany; 15 Institute of Pathology, University of Lübeck and University Medical Center Schleswig-Holstein, Campus Lübeck, Ratzeburger Allee 160, 23538 Lübeck, Germany; 16 Institute for Experimental Cancer Research, Kiel University (CAU) and University Medical Center Schleswig-Holstein, Campus Kiel, Arnold-Heller-Strasse 3, 24105 Kiel, Germany; 17 Institute of Medical Microbiology, Virology and Hygiene, University Medical Center Hamburg-Eppendorf, Hamburg, Germany; 18 Medical School Berlin, Leipziger Platz 10, 10117 Berlin, Germany

**Keywords:** Integrin β4, E-selectin, P-selectin, Myeloid-derived suppressor cell, Anoikis, Tumor-infiltrating leukocyte, Chemoattraction

## Abstract

**Background:**

The immunological composition of the tumor microenvironment has a decisive influence on the biological course of cancer and is therefore of profound clinical relevance. In this study, we analyzed the cooperative effects of integrin β4 (ITGB4) on tumor cells and E-/P-selectin on endothelial cells within the tumor stroma for regulating tumor growth by shaping the local and systemic immune environment.

**Methods:**

We used several preclinical mouse models for different solid human cancer types (xenograft and syngeneic) to explore the role of ITGB4 (shRNA-mediated knockdown in tumor cells) and E-/P-selectins (knockout in mice) for tumor growth; effects on apoptosis, proliferation and intratumoral signaling pathways were determined by histological and biochemical methods and 3D in vitro experiments; changes in the intratumoral and systemic immune cell composition were determined by flow cytometry and immunohistochemistry; chemokine levels and their attracting potential were measured by ELISA and 3D invasion assays.

**Results:**

We observed a very robust synergism between ITGB4 and E-/P-selectin for the regulation of tumor growth, accompanied by an increased recruitment of CD11b^+^ Gr-1^Hi^ cells with low granularity (i.e., myeloid-derived suppressor cells, MDSCs) specifically into ITGB4-depleted tumors. ITGB4-depleted tumors undergo apoptosis and actively attract MDSCs, well-known to promote tumor growth in several cancers, via increased secretion of different chemokines. MDSC trafficking into tumors crucially depends on E-/P-selectin expression. Analyses of clinical samples confirmed an inverse relationship between ITGB4 expression in tumors and number of tumor-infiltrating leukocytes.

**Conclusions:**

These findings suggest a distinct vulnerability of ITGB4^Lo^ tumors for MDSC-directed immunotherapies.

**Graphical Abstract:**

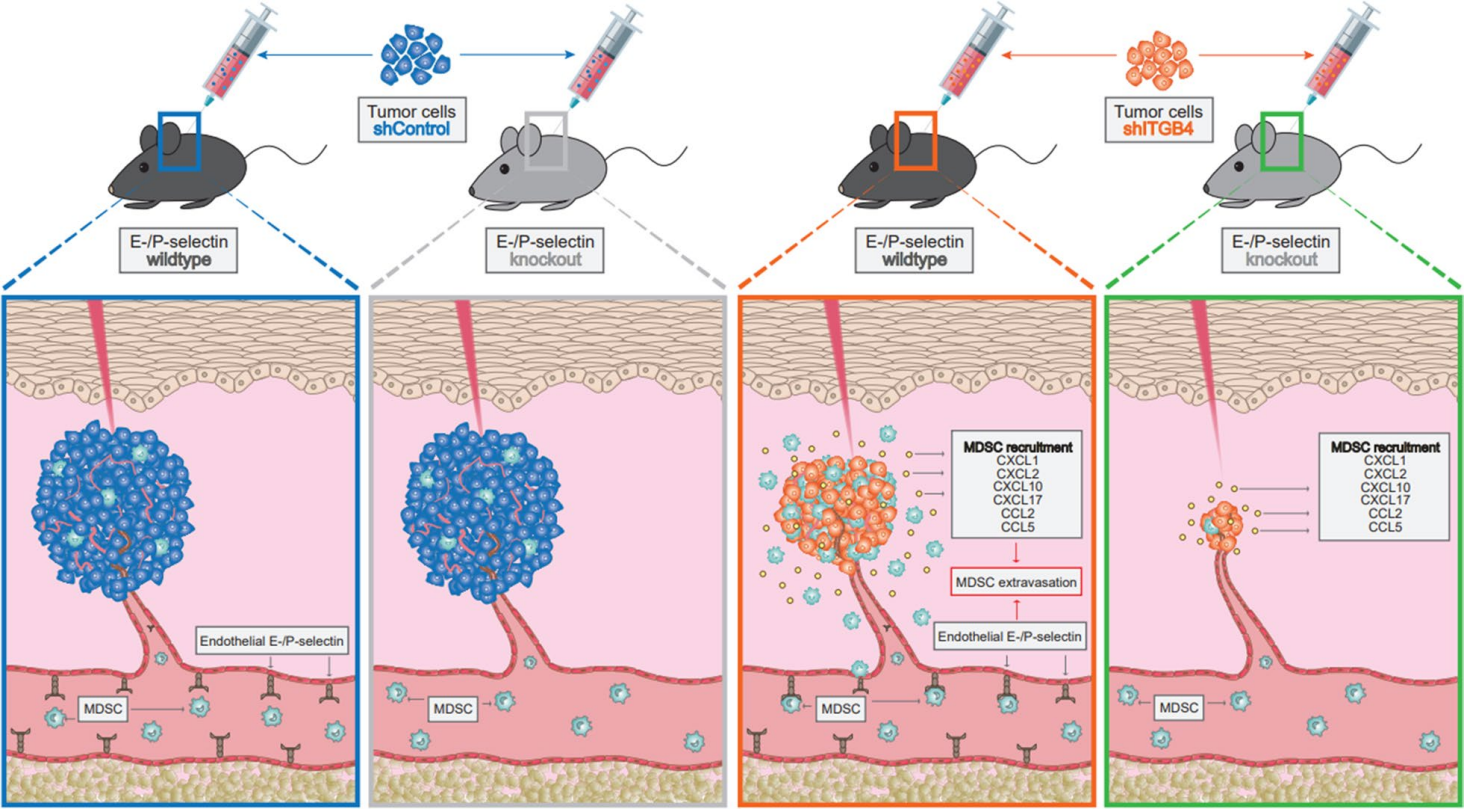

**Supplementary Information:**

The online version contains supplementary material available at 10.1186/s13045-023-01413-9.

## Background

Adhesion molecules are of paramount functional importance in cancer progression. Tumor cells (TCs) engage in mutual adhesive interactions with neighboring TCs, stromal cells, leukocytes, platelets and endothelial cells (ECs), to name a few, but also interact with components of the extracellular matrix (ECM), which collectively contribute to tumor progression and metastasis [1]. One rate-limiting step of hematogenous and intraperitoneal metastasis is the successful adhesion of circulating tumor cells (CTCs) to vascular ECs and of TCs in the abdominal fluid to peritoneal mesothelial cells, which provides the basis for subsequent transendothelial and mesothelial migration, respectively [2, 3]. In close analogy to the adhesion of leukocytes to ECs during inflammation, this adhesion process of TCs is also controlled by molecules of the leukocyte adhesion cascade, i.e., by selectin and integrin family members which may act in a functionally redundant fashion [4].

Selectins form a family of structurally and functionally related glycoproteins and two of its members, namely E- and P-selectin establish heterophilic cell contacts between TCs, platelets and ECs. E-selectin is an inducible transmembrane adhesion receptor on the luminal surface of ECs. With the exception of the smallest blood vessels of the skin and bone marrow (BM), where E-selectin is constitutively expressed [5, 6], it is transcriptionally induced by inflammatory cytokines, such as tumor necrosis factor-α and interleukin-1 [7]. This mainly transient expression of E-selectin serves the targeted recruitment of leukocytes to sites of inflammation. P-selectin exhibits partial functional redundancy to E-selectin [8, 9], but is not only expressed by ECs, but also activated platelets. ECs have special storage granules, the Weibel-Palade bodies, in which functional P-selectin is localized [10]. P-selectin can be mobilized from the granules to be subsequently expressed on the plasma membrane within a few minutes upon activation by inflammatory as well as hemostatic mediators such as thrombin, platelet-activating factor or histamine [10, 11]. Due to the property of rapid translocation, initial contact (capture, tethering) of leukocytes from the bloodstream is achieved via P-selectin [12].

Integrins are heterodimeric transmembrane adhesion receptors that enable cellular communication by mediating heterotypic cell–cell interactions and linkages of cytoskeletal components with the ECM. In addition to adhesion, integrins are crucial for a variety of cellular processes, including cell migration, proliferation, differentiation and survival [13]. Structurally, integrins consist of an α- and β-subunit of type I transmembrane glycoproteins that are linked to each other via non-covalent binding. So far, 18 α- and 8 β-subunits have been described, which can form 24 different heterodimeric integrin receptors by multiple combination [14]. In their capacity as bidirectional signal transducers, integrins can be activated not only by ligands within the ECM but also by intracellular signals (outside-in- and inside-out-signaling) [15]. While selectins mediate the aforementioned initial, rather weak contact between leukocytes and ECs [16], subsequent activating conformational changes of integrins enhance their affinity and avidity to their endothelial receptors, thereby mediating firm adhesion [17].

The role of E- and P-selectins for hematogenous and intraperitoneal metastasis is firmly established. Both the expression of E- and P-selectin ligands on the TC surface as well as the ability to adhere to E- and P-selectin have been described for various human carcinomas [18]. In spontaneous metastasis xenograft models of human breast [19], pancreatic [3], lung [20] and colon cancer [21], E- and P-selectin double deficiency drastically reduces spontaneous metastasis formation. However, this adhesion cascade appears to be redundant and the absence of selectin binding sites on cancer cells can be compensated for by other molecules of the leukocyte adhesion cascade [22] and in particular integrins [23]. In human prostate cancer xenografts, for instance, we observed that distant metastasis formation takes place equally well in E- and P-selectin double knockout (KO) mice, as reflected by a widespread absence of E-selectin binding sites in prostate cancer samples [24]. Based on the presumed relevance of the leukocyte adhesion cascade and its redundancy for distant metastasis formation, we next analyzed which integrins might be relevant—alone or in combination with E-/ P-selectin—for metastasis formation in prostate cancer xenografts. For this purpose, highly vs. weakly metastatic prostate cancer xenografts [25] were screened for integrin subunit expression as a first step of the present study.

## RESULTS

### ITGB4 is upregulated in highly metastatic prostate cancer cells

**Fig. 1:**
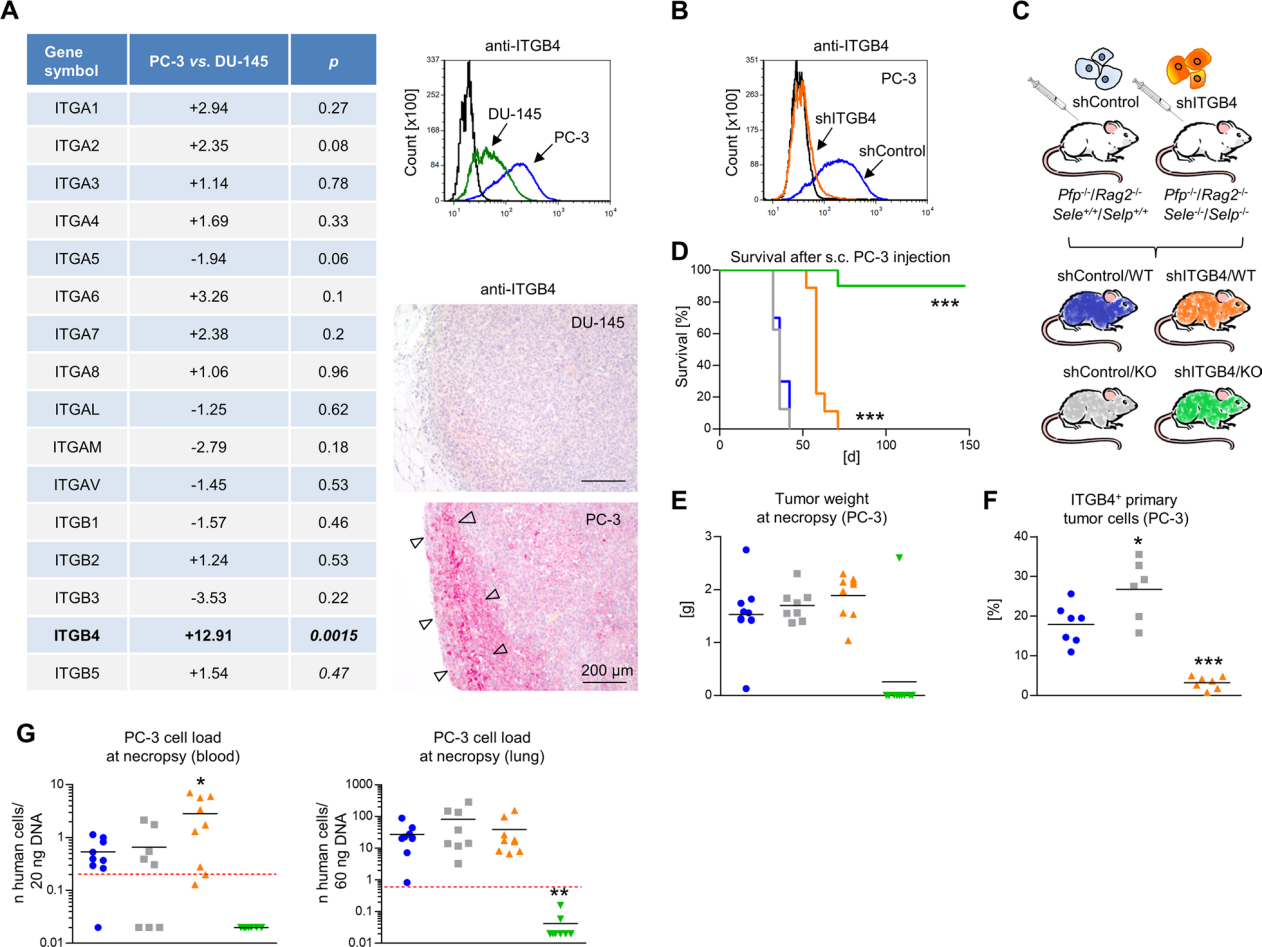
Combined depletion of ITGB4 and E-/P-selectin cooperatively reduces human prostate cancer xenograft tumor formation. Mean fold up- or down-regulation of integrin subunit gene expression comparing highly metastatic PC-3 vs. weakly metastatic DU-145 human prostate cancer cells (n = 3) and validation of differential ITGB4 protein levels in vitro and in situ*.* Arrowheads indicate ITGB4^+^ tumor cells at the xenograft tumor margin (**A**). shRNA-mediated knockdown of ITGB4 in PC-3 cells (**B**) and s. c. xeno-transplantation of shControl vs. shITGB4 cells into E-/P-selectin wildtype vs. knockout *Pfp*^−/−^/*Rag2*^−/−^ mice resulting in four experimental groups: shControl/WT (blue), shITGB4/WT (orange), shControl/KO (grey), shITGB4/KO (green) as illustrated (**C**). Survival of mice after s. c. injection of tumor cells (endpoint: s. c. xenograft tumor of ~ 1.5 cm^3^, **D**). Tumor weights (**E**) and percentage of ITGB4^+^ primary tumor cells (**F**) at necropsy. Human cell loads in blood and lung samples at necropsy (s. c. xenograft experiment) (**G**). Black lines in scatter plots represent mean values. Red dotted lines in (**G**) indicate the detection limit of the respective *Alu*-PCR experiment. All statistical comparisons indicated by asterisks were calculated vs. shControl/WT. *p < 0.05; **p < 0.01; ***p < 0.001

In our previous experiments using clinically relevant spontaneous metastasis xenograft models, we observed that the human prostate cancer cell line PC-3 has a notably higher metastatic potential than DU-145 prostate cancer cells in immunodeficient mice [24, 25]. Using qPCR profiler arrays, we revealed a significant upregulation of the integrin subunit beta 4 (ITGB4) in PC-3 as compared to DU-145 cells (Fig. 1A). This difference in the ITGB4 expression was further validated at the protein level in vitro and in the xenograft tumors in vivo (Fig. 1A). Interestingly, ITGB4 expression was enhanced at the tumor margin of PC-3 xenografts (Fig. 1A, arrowheads).

### ITGB4 knockdown in tumor cells and E-/P-selectin knockout in the tumor stroma synergistically delay human prostate cancer xenograft tumor formation in vivo

Given the putative redundancy of selectins and integrins in the leukocyte adhesion cascade, we next aimed to analyze whether ITGB4 alone or in combination with E-/ P-selectins plays a role for spontaneous metastasis formation in the PC-3 xenograft model. For this purpose, we generated a shRNA-mediated knockdown (KD) of ITGB4 in PC-3 cells (Fig. 1B) and carried out spontaneous metastasis experiments with these derivative cell lines. We either injected immunodeficient *Pfp*^−/−^/ *Rag2*^−/−^ mice with E-/P-selectin expression (WT) or E-/P-selectin knockout (KO) (*Sele*^−/−^/*Selp*^−/−^) mice subcutaneously (s.c.) with control transfected (shControl) or ITGB4 KD (shITGB4) TCs, resulting in the four experimental groups as illustrated in Fig. 1C. The endpoint of the experiment was a primary tumor size of ~ 1.5 cm^3^ with the aim to quantify distant metastatic cell loads at this time point. The experiment was also terminated in case of skin ulceration above the growing primary tumor. To our surprise, ITGB4 KD alone already notably delayed s.c. xenograft tumor formation: while the time span from tumor engraftment to endpoint was nearly similar in the shControl/WT and shControl/KO groups (36.6 ± 4.1 days *vs*. 35.3 ± 3.4 days), the shITGB4/WT group showed a significant tumor growth delay (59.3 ± 5.2 days). Most strikingly, however, tumor formation was almost entirely abolished after combining the ITGB4 KD with the E-/P-selectin KO. Only one of ten mice of the combination group developed a primary tumor reaching the endpoint after 72 days. All other mice did not develop detectable tumors until the experiment was terminated on day 147 (Fig. 1D). The xenograft tumor weights in the different groups at necropsy are shown in Fig. 1E. Stability of the ITGB4 KD after in vivo growth was validated by ex vivo flow cytometry of the resected primary tumor tissue (Fig. 1F). Interestingly, the percentage of ITGB4^+^ primary TCs increased in the shControl/KO group (Fig. 1F). At necropsy, the human cell load in the blood was elevated in the shITGB4/WT group in comparison with the shControl/WT group, while the human cell load in the lung was not altered (Fig. 1G). In accordance with the widespread absence of primary tumors, human cells were neither detectable in the blood nor in the lungs of mice from the shITGB4/KO group (Fig. 1G).

### ITGB4 knockdown prevents formation of disseminated tumor deposits after i.v. injection

Given the lack of primary tumor growth, a potential cooperative role of E-/P-selectins and ITGB4 for extravasation could principally not be explored in the s.c. xenograft model. To this end, our observations suggested an impaired extravasation capacity of ITGB4 KD TCs as the human cell load in the blood, but not in the lung, was enhanced upon KD in WT mice. Therefore, we next applied an intravenous (i.v.) dissemination model and observed the formation of intrathoracic, intra-abdominal (visceral and parietal) and musculoskeletal macrometastases two months after injection in 100% of the shControl/WT group, 50% of the shControl/KO group and 0% of both shITGB4 groups (Additional file 1: Fig. S1). At the level of the total human DNA per tissue DNA template (*Alu*-PCR), metastatic cells were mainly present in the lungs of shControl/WT and shControl/KO mice, but not in the lungs of mice from the two shITGB4 groups (Additional file 1: Fig. S1), indicating that ITGB4 is required for the formation of disseminated tumor deposits after i.v. administration.

### ITGB4 and E-/P-selectin synergistically delay human pancreatic cancer xenograft tumor growth in vivo

**Fig. 2:**
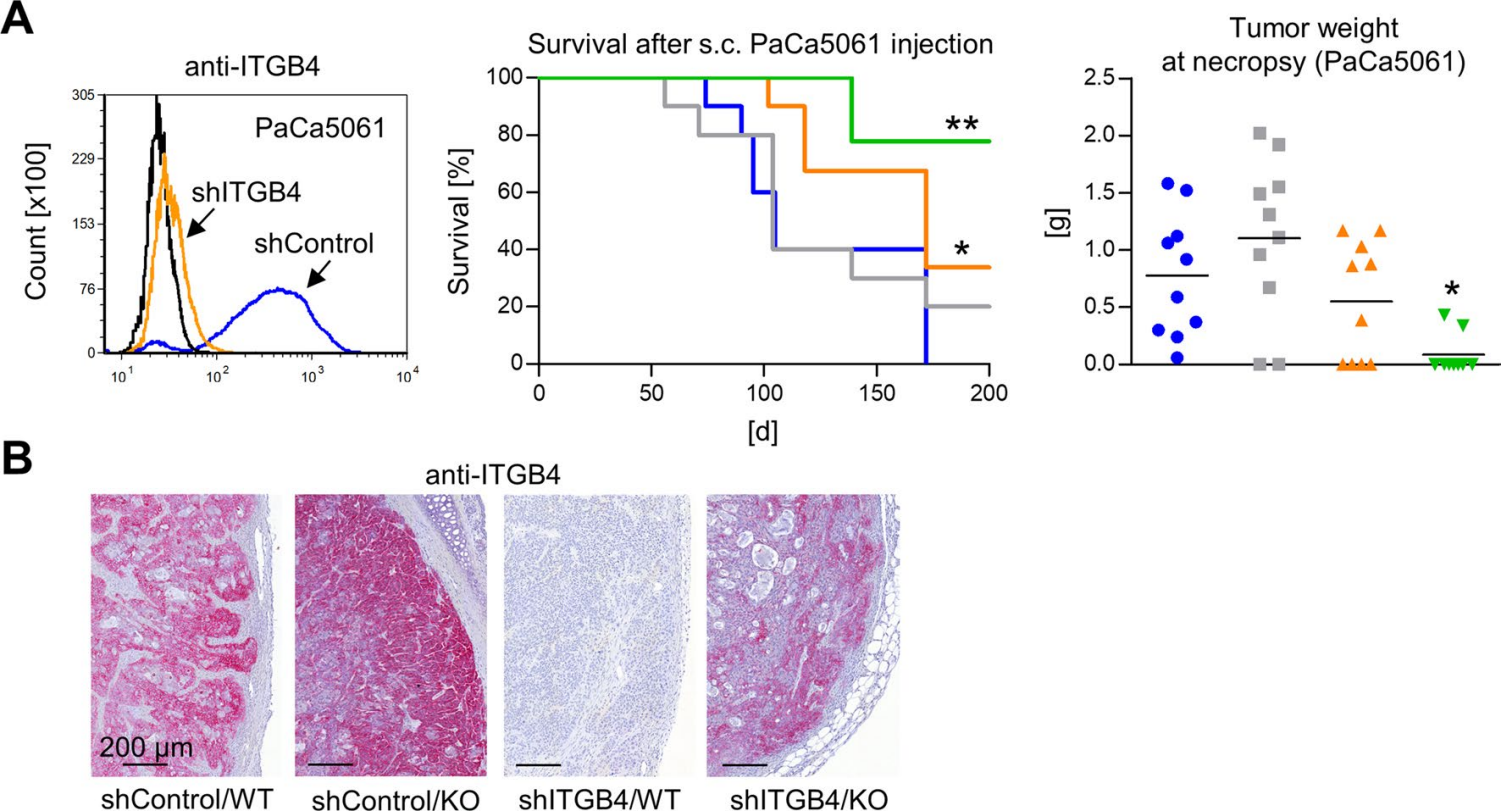
Combined depletion of ITGB4 and E-/P-selectin cooperatively reduces human pancreatic cancer xenograft tumor formation*.* shRNA-mediated knockdown of ITGB4 in PaCa5061 cells, survival of mice after s. c. xeno-transplantation of shControl vs. shITGB4 cells into E-/P-selectin wildtype vs. knockout *Pfp*^−/−^/*Rag2*^−/−^ mice (endpoint: tumor size ~ 1.5 cm^3^) and resulting tumor weights at necropsy (**A**). Representative anti-ITGB4 immunostaining images taken from s. c. PaCa5061 xenograft tumors (**B**). All statistical comparisons indicated by asterisks were calculated vs. shControl/ WT. *p < 0.05; **p < 0.01

In the next step, we aimed to validate our most striking observation, i.e., the synergistic and almost complete reduction of xenograft tumor formation upon combined ITGB4 KD and E-/P-selectin KO by means of additional ITGB4-expressing xenograft models. As seen for PC-3 tumors, KD of ITGB4 in human pancreatic cancer cells (PaCa5061) significantly impaired s.c. xenograft tumor growth in WT *Pfp*^−/−^/*Rag2*^−/−^ mice (Fig. 2A). Only six out of ten mice from the shITGB4/WT group developed tumors (155.9 ± 40.8 days) as opposed to ten out of ten mice in the shControl/WT group (125.2 ± 41.2 days) (Fig. 2A). E-/P-selectin KO alone had no significant effect on the survival of the mice that were injected with shControl cells: eight of ten mice developed tumors after 125.4 ± 50.7 days (Fig. 2A). Again, the combination of ITGB4 KD and E-/P-selectin KO most strikingly reduced tumor formation: only two out of nine mice developed tumors after 186.4 ± 26.9 days (Fig. 2A). The experiment was terminated on day 200 post injection. Stable ITGB4 KD was validated by immunohistochemistry (IHC) of primary tumors (Fig. 2B). In the two available tumors of the combination group the ITGB4 KD was not as efficient as in the shITGB4/WT group (Fig. 2B). Hence, ITGB4 again appeared to be specifically up-regulated in the E-/P-selectin-deficient environment.

### ITGB4 and E-/P-selectin synergistically delay human ovarian cancer xenograft tumor growth in vivo

**Fig. 3:**
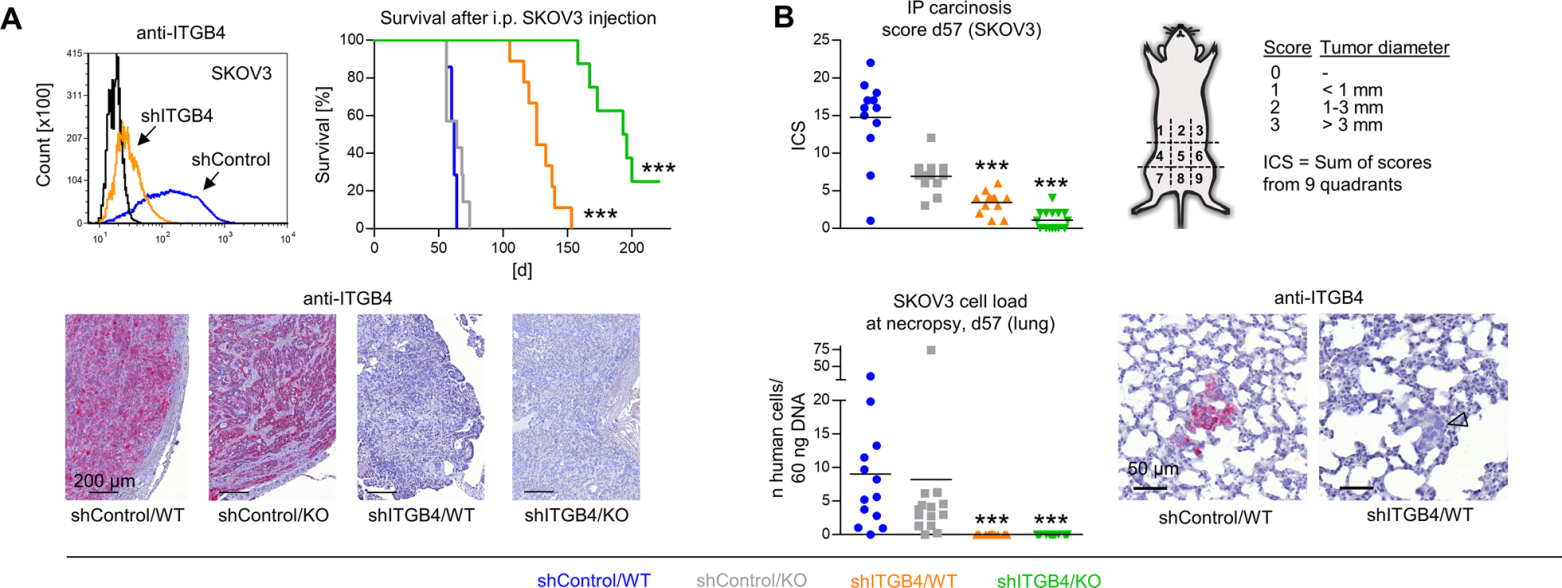
Combined depletion of ITGB4 and E-/P-selectin cooperatively reduces human ovarian cancer xenograft tumor formation*.* shRNA-mediated knockdown of ITGB4 in SKOV3 cells, survival of mice after i. p. injection of shControl vs. shITGB4 cells into E-/P-selectin wildtype vs. knockout SCID mice (endpoint: palpable abdominal tumor masses or ascites) and representative anti-ITGB4 immunostaining images from i. p. SKOV3 xenograft tumors (**A**). Intraperitoneal carcinosis score (ICS) determined as illustrated and spontaneous pulmonary metastatic cell load determined by *Alu*-PCR on day 57 after i. p. injection of SKOV3 shControl vs. shITGB4 cells into E-/P-selectin wildtype vs. knockout SCID mice (**B**). Representative anti-ITGB4 immunostaining images of spontaneous SKOV3 lung metastases (arrowhead indicates an ITGB4^−^ lung metastasis) (**B**). All statistical comparisons indicated by asterisks were calculated vs. shControl/ WT. ***p < 0.001

In addition, we aimed to explore whether similar observations could be made after using a different TC application route in a different immunodeficient mouse strain. In an intraperitoneal (i.p.) carcinomatosis xenograft model, in which human ovarian cancer cells (SKOV3) were i.p. injected into SCID mice, the same synergism of ITGB4 KD and E-/P-selectin KO could be observed (Fig. 3A). Similarly, E-/P-selectin KO alone had no effect on mouse survival (63.3 ± 7.4 days as compared to 61.1 ± 2.8 days in the shControl/WT group), ITGB4 KD had a moderate effect (128.6 ± 14.3 days) and the combination of both strikingly improved mouse survival (191.2 ± 23.6 days, only six of eight mice with tumors, termination on day 221). Stable ITGB4 KD was validated by IHC of injection site tumors (Fig. 3A, lower row). In case of the SKOV3 model, ITGB4 was not up-regulated in E-/P-selectin KO mice. By performing an additional endpoint experiment (Fig. 3B), we specifically determined i.p. carcinosis formation according to the scheme in Fig. 3B in the four experimental groups after similar time spans (57 days). Reflecting the outcome differences in the survival experiment, we observed a significant reduction of the intraperitoneal carcinosis score (ICS) in shITGB4/WT and even more pronounced in shITGB4/KO mice (Fig. 3B). Besides i.p. carcinosis, we analyzed hematogenous lung metastasis formation of the i.p. xenografts and found that the pulmonary metastatic load was markedly reduced in both shITGB4 groups. The few remaining lung metastases showed stable ITGB4 depletion as determined by IHC (Fig. 3B, lower row, arrowhead).

The survival data from all three xenograft models were analyzed using Cox proportional hazards regression models which included cell line and KD condition as predictors to obtain cell line-adjusted effects of the ITGB4 KD condition (Additional file 2: Fig. S2). Adjusted for the cell line, there was a significant beneficial effect of the ITGB4 KD on mouse survival (HR: 0.12), which was strikingly increased when combined with the E-/P-selectin KO (HR: 0.01). In contrast, the E-/P-selectin KO condition alone did not improve survival (HR: 0.78).

### Effects of ITGB4 depletion on the percentage of apoptotic and proliferative xenograft tumor cells in situ

**Fig. 4:**
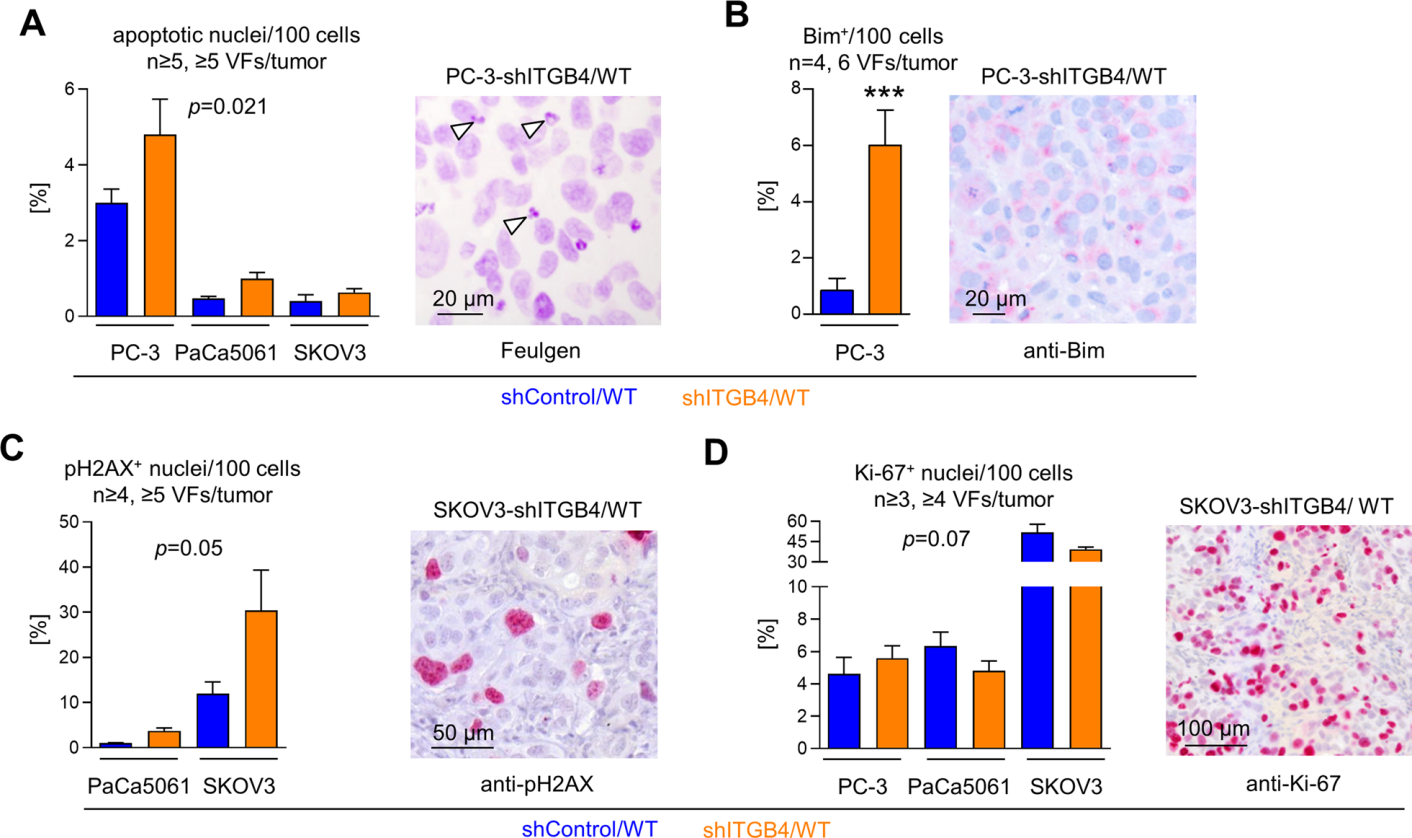
Depletion of ITGB4 causes increased apoptosis, but unaltered proliferation in vivo. Percentage of apoptotic nuclei (**A**), Bim^+^ tumor cells (**B**), pH2AX^+^ nuclei (**C**) and Ki-67^+^ nuclei (**D**) per viewing field (VF) in xenograft tumor tissues and corresponding representative staining microphotographs (arrowheads in (**A**) indicate apoptotic bodies). Bar charts represent mean + SD of given sample sizes. ***p < 0.001

In a first attempt to explain our in vivo observations, we hypothesized that the growth retardation upon KD of ITGB4, an essential component of the hemidesmosome which suppresses anoikis [26], can be explained by enhanced apoptosis. Feulgen DNA staining demonstrated higher amounts of apoptotic bodies in shITGB4 compared to shControl tumors in all three tumor entities (cell line-adjusted effect: 0.79, 95% CI: 0.15–1.4], *p* = 0.021, Fig. 4A). At the molecular level, we could substantiate this observation by means of the apoptosis markers Bim in case of PC-3 xenografts (5.17, 95% CI: 2.46–7.88, *p* < 0.001, Fig. 4B) and pH2AX in case of PaCa5061 as well as SKOV3 xenografts (9.62 [1.01; 18.23], *p* = 0.05, Fig. 4C). In contrast, Ki67 staining revealed no difference in the amount of proliferative xenograft TCs between shControl and shITGB4 xenograft tumors in all three models (− 3.46 [− 6.98; 0.06], *p* = 0.07, Fig. 4D). Thus, growth retardation upon ITGB4 KD appeared to be due to enhanced apoptosis.

### Effects of ITGB4 depletion on intratumoral signaling molecule levels

To identify possible signaling pathways that could underlie the observed changes in tumor growth, Western blot (WB) analyses were initially carried out only with xenograft tumor lysates of the PC-3 model as a first step. Among several tested cell cycle-, survival- and ITGB4-related molecules, we identified a RAF-/MEK-independent activation (phosphorylation) of p42/44 MAPK upon ITGB4 KD whereas the p42/44 MAPK:p38MAPK ratio was decreased in this group (Additional file 3: Fig. S3). The p42/44 MAPK level was specifically increased in the shControl/KO group. In addition, pSRC and pJNK were decreased in both the shControl/KO and shITGB4/WT group as compared to the shControl/WT group. The cyclin D3 and p27 protein levels were elevated in the ITGB4 KD group (Additional file 3: Fig. S3). Tumors from the combination group (shITGB4/KO) could not be included in the analysis due to the almost complete inhibition of tumor growth.

### Effects of ITGB4 depletion on cell proliferation, three-dimensional colony forming capacity and apoptosis in vitro

**Fig. 5:**
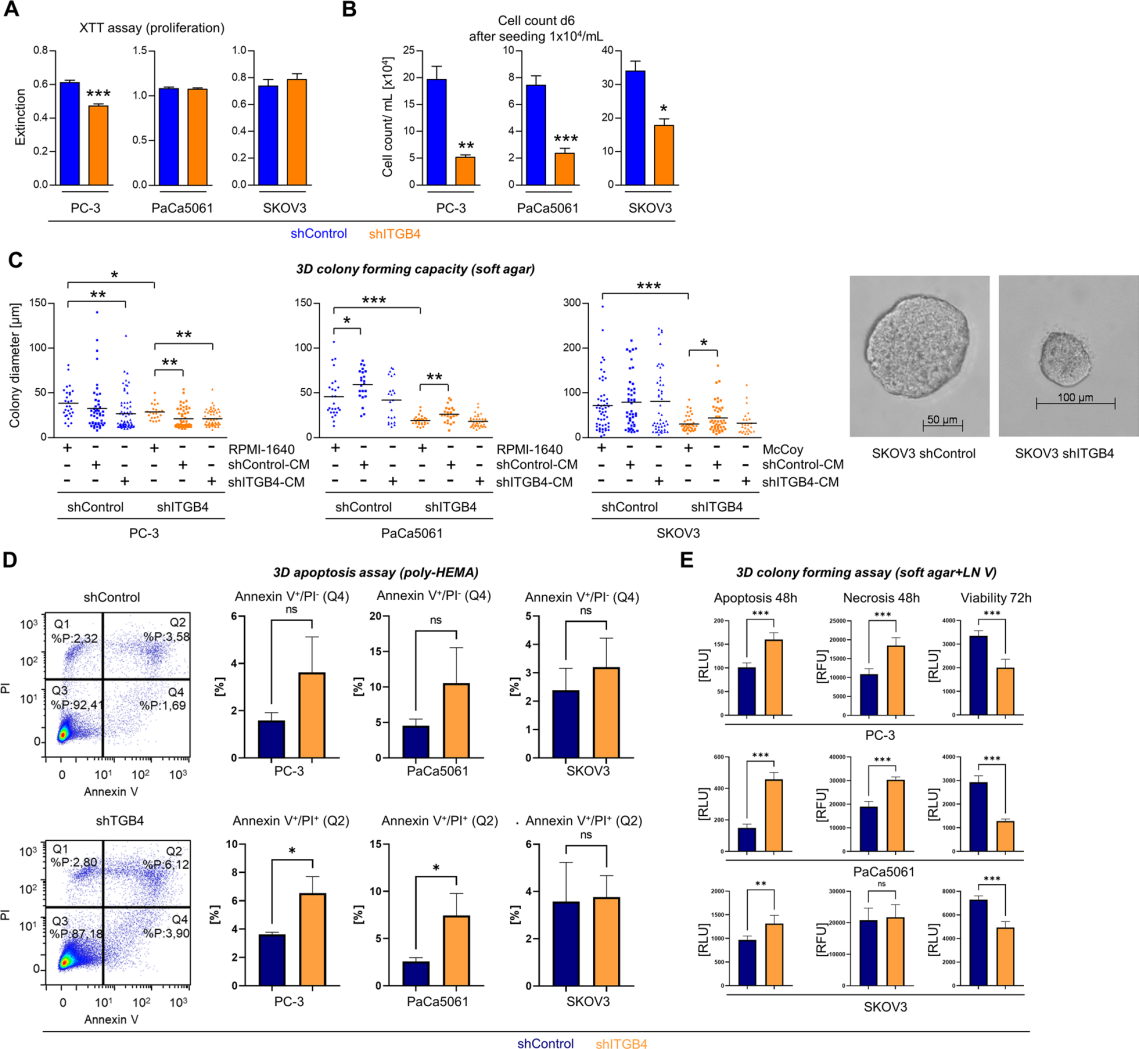
Depletion of ITGB4 reduces proliferation at low confluence, impairs three-dimensional colony forming capacity and enhances apoptosis in vitro. Extinction values in colorimetric proliferation XTT assays at high cell density in conventional cell culture (**A**). Cell counts per mL on day six after low-density seeding in conventional cell culture (**B**). Diameters of 3D tumor spheroids after eleven days of growth in soft agar in the presence of standard cell culture media or conditioned media (CM) taken from shControl or shITGB4 cell cultures (**C**). Percentage of early (Q4) and late (Q2) apoptotic cells in 3D tumor spheroids developed after three days in poly-HEMA-treated cell culture flasks (**D**). Luminescence and fluorescence measurements indicating the amount of apoptotic, necrotic and viable cells at indicated time points after cultivation in soft agar containing laminin V (LN V, laminin-α3β3γ2) (**E**). Bar charts represent mean + SD of n = 3. Lines in scatter plots indicate mean values. *p < 0.05; **F < 0.01; ***p < 0.001

To investigate the effects of the ITGB4 KD on the TC behavior in further detail, we next assessed cell proliferation in conventional 2D cell culture, the three-dimensional (3D) colony forming capacity in soft agar as well as apoptosis induction under anchorage-independent growth conditions. To simulate the high cell density in late stage tumors, XTT assays were performed at high confluence. By this approach, we observed hardly any to only marginal differences in the proliferative activity of shControl vs. shITGB4 cells (Fig. 5A). To mimic the situation in the early phase of tumor establishment, we measured the cell counts six days after low confluence seeding. Here, shITGB4 cells were less proliferative than shControl cells (Fig. 5B). Soft agar assays revealed that the ITGB4 KD consistently reduced the 3D colony forming capacity in all three tested models (cultured with fresh cell culture media, Fig. 5C). This finding resembled the in vivo situation very well. To test whether secreted factors within the conditioned media (CM) of shControl or shITGB4 TC cultures rescue the colony formation capacity particularly of shITGB4 cells, the soft agar assays were additionally performed using corresponding shControl-CM or shITGB4-CM. The addition of CM, however, caused inconsistent and divergent effects on the different tested models (Fig. 5C). Therefore, the limitation of colony formation ability was not consistently due to an ITGB4-dependent release of soluble factors.

Next, 3D colonies generated under anchorage-independent growth conditions in poly-HEMA-coated flasks were separated into single-cell suspensions and subjected to flow cytometric apoptosis assays (representative density plots for PC-3 cells shown in Fig. 5D). Thereby, a higher percentage of late (Annexin V^+^/ PI^+^, Q2) apoptotic cells could be observed for shITGB4 as compared to shControl cells in the PC-3 and PaCa5061 models, but not with SKOV3 cells (Fig. 5D). In soft agar assays containing the major ITGB4 ligand laminin V (LN V, laminin-α3β3γ2), the amount of apoptotic and necrotic cells was significantly increased in shITGB4 as compared to shControl cells in all tested models at 48 h while their viability at 72 h was significantly decreased (Fig. 5E). Thus, ITGB4 promotes colony formation in 3D conditions by suppressing apoptosis particularly in the presence of its main ligand laminin-V.

### E-selectin, P-selectin and ITGB4 cooperate to regulate tumor growth and ITGB4 depletion is accompanied by enhanced leukocyte infiltration into xenograft tumors

**Fig. 6:**
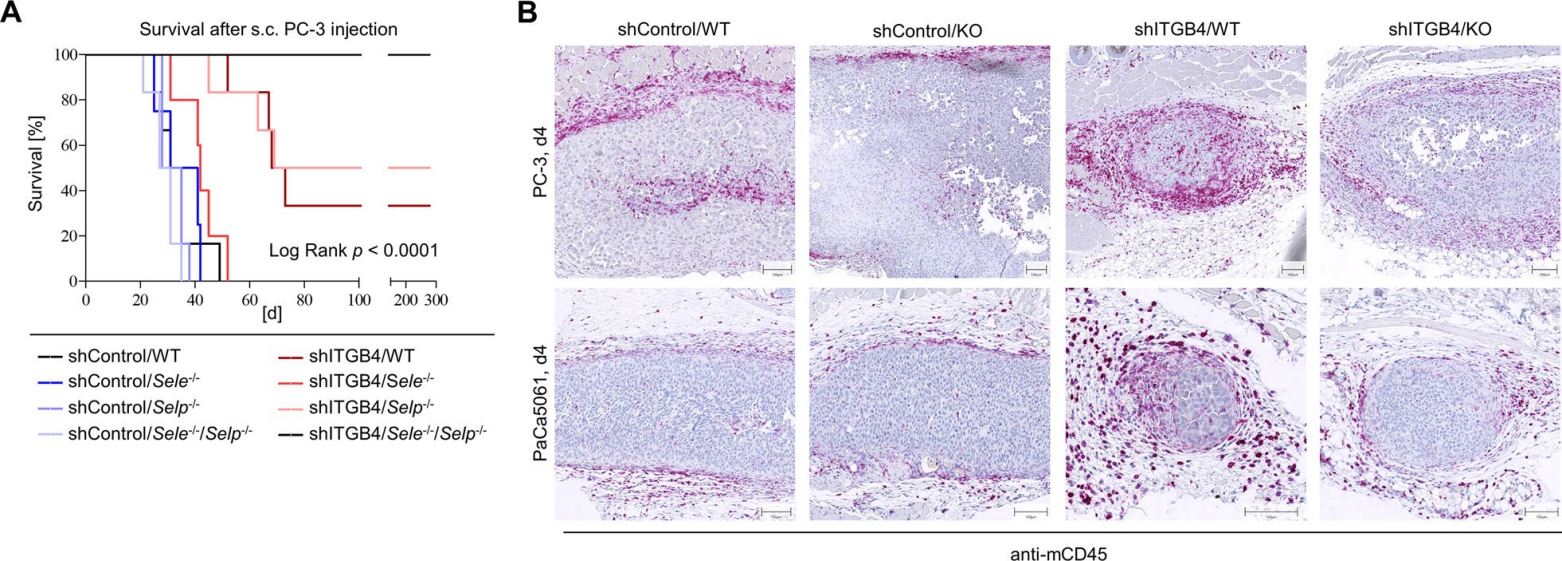
ITGB4 depletion requires E- and P-selectin double knockout for synergistic tumor growth reduction and is accompanied by increased leukocyte infiltration. Mouse survival after s. c. injection of PC-3 control vs. ITGB4 knockdown cells into E-/P-selectin wildtype, single or double knockout mice (endpoint: xenograft tumor size ~ 1.5 cm^3^) (**A**). Representative anti-mCD45 immunostaining images of initial tumor nodules on day 4 after s. c. injection of PC-3 or PaCa5061 control or ITGB4 KD cells into E-/P-selectin wildtype or double knockout mice (**B**)

In the next step, we aimed to clarify whether the observed cooperation between ITGB4 and selectins depends on either E- or P-selectin alone or on both molecules. For this purpose, a further survival experiment with PC-3 shControl and shITGB4 cells was carried out in *Pfp*^−/−^/*Rag2*^−/−^ mice with E- and/or P-selectin single or double KO. Importantly, a cooperatively reduced tumor formation was only detectable when shITGB4 cells were injected into E-/P-selectin double KO mice. This effect was again very stable; there was no detectable tumor growth in the shITGB4/*Sele*^−/−^/*Selp*^−/−^ group when the experiment was terminated on day 279 (Fig. 6A). The E-/P-selectin double KO alone had again no effect on shControl cells. The ITGB4 KD per se again had a detectable, but less striking effect on tumor growth (Fig. 6A).

Based on the literature, phenotypic differences in E- and P-selectin double-deficient as opposed to single-deficient mice have been particularly described with regard to leukocyte emigration in the context of inflammation [27]. Therefore, we hypothesized that the synergism visible only in E-/P-selectin double KO mice might rely on impaired leukocyte migration into the developing tumors, assuming that tumor-infiltrating leukocytes have pro-tumorigenic properties [28] and may be particularly essential for the establishment of ITGB4 KD tumors. To test this hypothesis, PC-3 and PaCa5061 shControl and shITGB4 cells were injected into WT or E-/P-selectin-deficient mice and initial tumor nodules were harvested on day 4. Based on immunostainings for mCD45, the initial tumor nodules of the shITGB4/WT groups showed a higher amount of infiltrating mCD45^+^ cells compared to the other experimental groups (including the shITGB4/KO group), where mCD45^+^ cells were mainly located in the tumor periphery (Fig. 6B). The morphology of these leukocytes is shown in the HE stainings in Additional file 4: Fig. S4A. In PC-3 tumor nodules harvested on day 8 after injection, the enhanced leukocyte infiltrate in ITGB4 KD tumors was again visible, characterized by arginase-1- and myeloperoxidase (MPO)-positive cells. Inducible nitric oxide synthase (iNOS) stainings revealed a remarkable increase in fibroblast-like cells in ITGB4 KD tumors (Additional file 4: Fig. S4B). In a further in vivo experiment with SKOV3 cells which were injected i.p. into *Rag2*^−/−^ BALB/c mice, in which the synergism between ITGB4 KD and E-/P-selectin KO was again visible by means of the ICS, the malignant ascites of mice from the shITGB4/WT group contained notably more mCD45^+^ cells than that from the shControl/WT group (Additional file 4: Fig. S4C).

### ITGB4 knockdown enhances the attraction of human macrophages, chemokine secretion, and microvesicle release in vitro

**Fig. 7:**
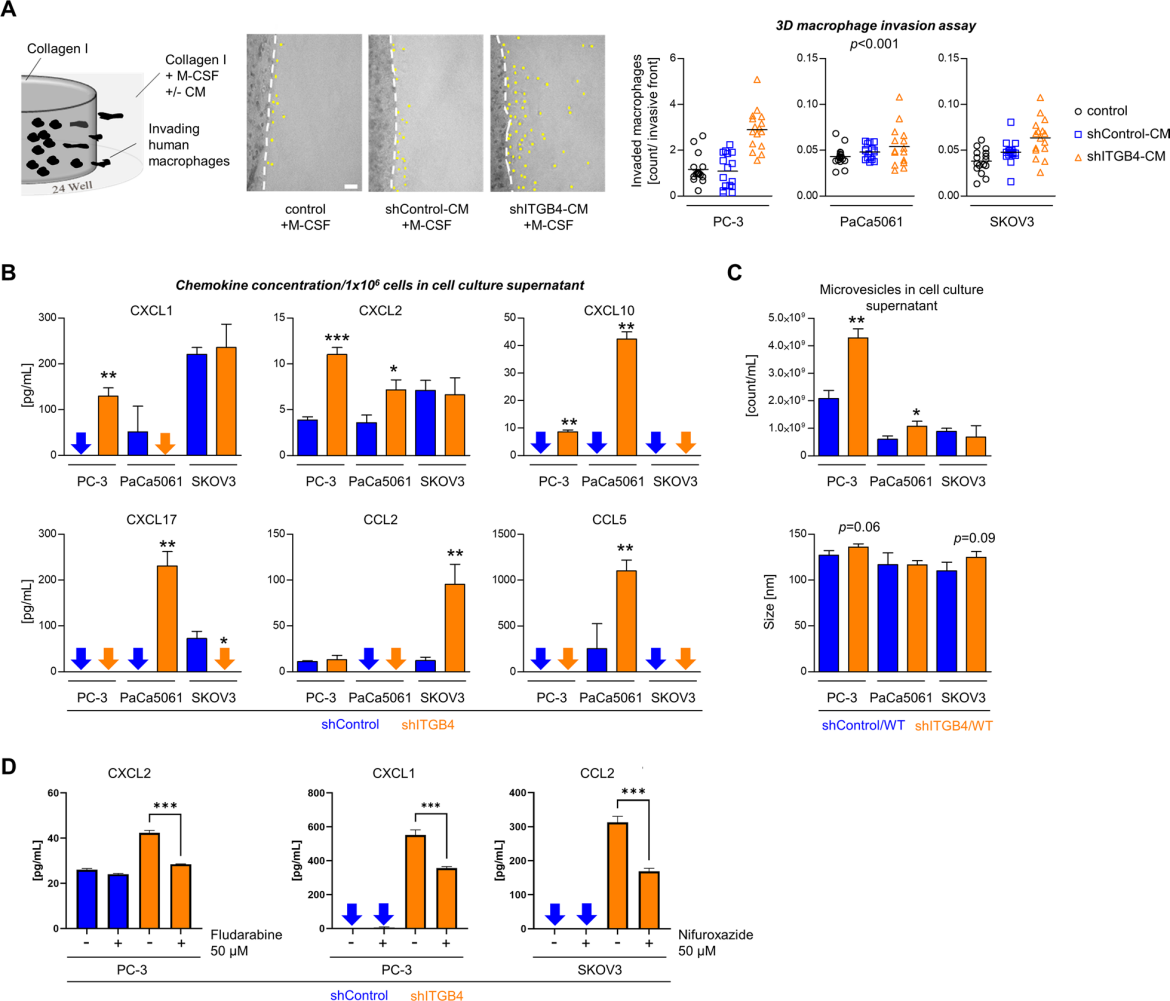
ITGB4 depletion enhances leukocyte attraction via chemokine release. Illustration of the experimental setup and quantification of invaded human macrophages per invasive front in a 3D collagen I matrix mixed with standard cell culture media or conditioned media (CM) taken from shControl or shITGB4 cell cultures, each supplemented with M-CSF (**A**). Concentrations of indicated chemokines in the supernatant of 1 × 10^6^ control or ITGB4 KD cells of the indicated cell lines (**B**). Number of microvesicles per mL and microvesicle size in supernatants of control or ITGB4 KD TC cultures (**C**). Concentrations of indicated chemokines in the supernatant of control or ITGB4 KD cells of the indicated cell lines with or without treatment with STAT inhibitors as indicated (**D**). Black lines in scatter plots indicate mean values. Bar charts represent mean + SD of n ≥ 3. *p < 0.05; **p < 0.01; ***p < 0.001

These findings suggested that the ITGB4 KD leads to a stronger immune cell infiltration of the tumor microenvironment (TME). To investigate whether leukocyte attraction is actively driven by the ITGB4 KD and even relevant in the human context, we tested the invasion behavior of human macrophages in a 3D spherical invasion assay in the presence or absence of CM from either shControl or shITGB4 TCs (as illustrated in Fig. 7A) [29]. We noticed that CM of shITGB4 TCs leads to an increased number of invading human macrophages compared to shControl CM or native medium in all three models (Fig. 7A, expected marginal mean averaged over cell line is 0.675, 95% CI: 0.441–0.909, *p* < 0.001 and 0.717, 95% CI: 0.482–0.951, *p* < 0.001 for the comparison to shControl-CM and control, respectively). This finding could be corroborated using macrophages isolated from different human donors (Additional file 5: Fig. S5) and supported the hypothesis that the ITGB4 KD is beneficial for the attraction of leukocytes.

Leukocyte recruitment into tumors by TCs themselves can be induced by the secretion of different chemokines. To investigate putative factors causing leukocyte attraction in ITGB4 KD TCs, we measured chemokine levels in TC supernatants using ELISA. We observed enhanced levels of CXCL1, CXCL2 and CXCL10 in cell culture supernatants of shITGB4 PC-3 TCs, increased amounts of CXCL2, CXCL10, CXCL17 and CCL5 in supernatants of shITGB4 PaCa5061 cells and elevated secretion of CCL2 in supernatants of shITGB4 SKOV3 cells (Fig. 7B). To further investigate whether, in addition to chemokine secretion, vesicular transport processes were affected by ITGB4 KD, we isolated microvesicles from shControl and shITGB4 TC supernatants. Nanoparticle tracking analysis (NTA) revealed no significant difference in microvesicle size, but significant variations in vesicle numbers (Fig. 7C). Supernatants from PC-3 and PaCa5061 shITGB4 cells contained approximately twice as many vesicles as respective shControl supernatants. This drastic difference was not observed with cell culture supernatants from SKOV3 shControl and shITGB4 TCs.

In the next step, we further explored the mechanism underlying the increased chemokine release upon ITGB4 KD. Interestingly, the enhanced release of CXCL2 from ITGB4 KD PC-3 cells could be reversed by treating the tumor cells with the STAT inhibitor fludarabine. Likewise, treating PC-3 and SKOV3 cells with the STAT inhibitor nifuroxazide notably impaired the induction of CXCL1 and CCL2 release upon ITGB4 KD, respectively (Fig. 7D).

### ITGB4 depletion specifically recruits CD11b^+^ Gr-1^+^ leukocytes into developing tumors and recruitment depends on host E-/P-selectin expression

**Fig. 8:**
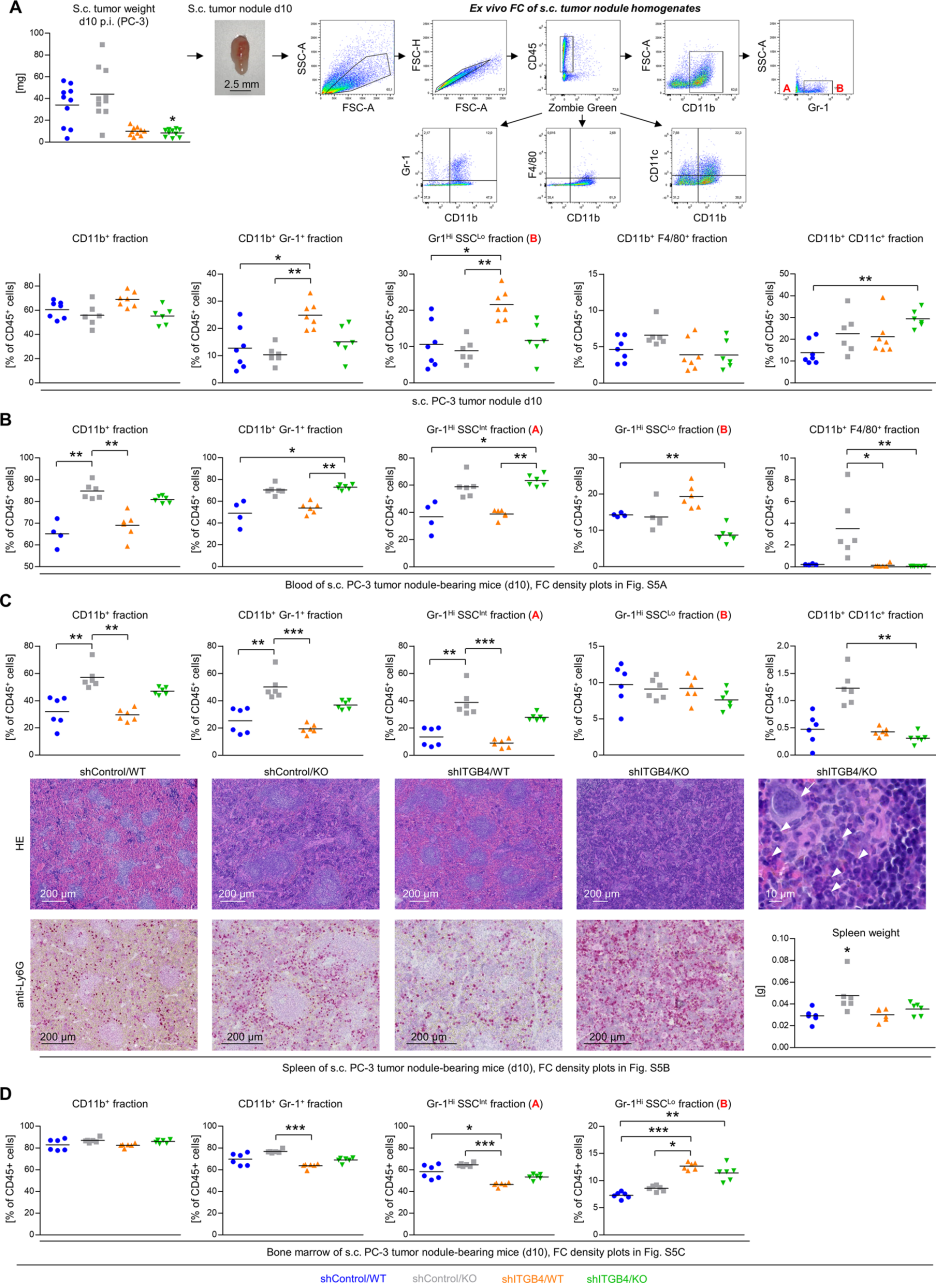
ITGB4 knockdown and E-/P-selectin knockout cooperatively determine the local and systemic immune environment in the early phase of tumor establishment. Tumor weights on d10 after s. c. injection of PC-3 control vs. ITGB4 knockdown cells into E-/P-selectin wildtype vs. knockout mice and representative density plots of the ex vivo flow cytometric analysis of s. c. tumor nodule homogenates (**A**). Fractions of leukocyte subpopulations characterized by indicated surface molecules and granularity (SSC) in the s. c. tumor nodules (**A**) and corresponding blood (**B**), spleen (**C**), and bone marrow (**D**) samples. For representative flow cytometry density plots of blood, spleen and bone marrow samples, please see Additional file 6: Fig. S6. Representative photomicrographs of HE- and anti-Ly6G (Gr-1 epitope)-stained spleen samples and corresponding spleen weights of s. c. tumor nodule-bearing mice (d10) (**C**, the arrow indicates a megakaryocyte, arrowheads indicate immature granulocytes with ring-shaped nuclei, see Additional file 7: Fig. S7 also). Black lines in scatter plots in (**A**)–(**D**) represent mean values. *p < 0.05; **p < 0.01; ***p < 0.001

The next step was to clarify whether a certain leukocyte subpopulation is specifically recruited into developing ITGB4 KD (as opposed to shControl) tumors and whether this recruitment is impaired in E-/P-selectin KO mice. Using multicolor flow cytometry, we analyzed the immune cell composition of s.c. xenograft tumor tissues ten days after inoculation. Tumor weights and the gating strategy are depicted in Fig. 8A. Briefly, viable CD45^+^ leukocytes were distinguished into neutrophils (CD11b^+^ Gr-1^+^), macrophages (CD11b^+^ F4/80^+^) and dendritic cells (CD11b^+^ CD11c^+^). CD11b^+^ myeloid cells were further subdivided into two populations based on Gr-1 expression and cellular granularity (side scatter, SSC): Gr-1^−^ SSC^Lo^ (A in Fig. 8A) and Gr-1^+^ SSC^Lo^ neutrophil progenitor cells (B in Fig. 8A). In shITGB4 tumors of WT mice we detected increased levels of CD11b^+^ Gr-1^+^ cells that comprised mainly Gr-1^+^ SSC^Lo^ cells. Most importantly, this increase was not observable in shITGB4 tumors of E-/P-selectin KO mice. In addition, the fraction of intratumoral CD11b^+^ CD11c^+^ dendritic cells was specifically increased in the shITGB4/KO group (Fig. 8a).

In parallel, we elucidated the immune cell composition in peripheral blood, spleen and BM during the early tumor establishment phase. Gating strategies for the different tissues are shown in Additional file 6: Figure S6. The peripheral blood samples of tumor-bearing E-/P-selectin KO mice contained a higher fraction of CD11b^+^ and CD11b^+^ Gr-1^+^ cells which were mainly related to the Gr-1^Hi^ SCC^Int^ (more differentiated neutrophils) population (Fig. 8B, see population A in Additional file 6: Fig. S6A also). This observation was largely independent of the TCs’ ITGB4 status. Notably, blood samples of shITGB4 tumor-bearing WT mice contained by trend elevated levels of Gr-1^+^ SSC^Lo^ cells (see population B in Suppl Fig. S6A), which was strikingly abolished when the mice had an additional E-/P-selectin KO (Fig. 8B). Moreover, the blood of E-/P-selectin KO mice bearing shControl tumors contained a higher fraction of CD11b^+^ F4/80^+^ macrophages compared to both shITGB4 groups (Fig. 8B).

The intrasplenic percentages of CD11b^+^ and CD11b^+^ Gr-1^+^ cells were increased in shControl/KO mice, which were related to the Gr-1^Hi^ SSC^Int^ population (Fig. 8C, see population A in Additional file 6: Fig. S6B also). In addition, we detected a significant reduction in the percentage of CD11b^+^ CD11c^+^ dendritic cells in the spleens of mice from the shITGB4/KO group as compared to those from the shControl/WT group (Fig. 8C). As previously reported by other groups [8], we noticed splenic enlargement in E-/P-selectin KO mice (Fig. 8C). HE and Ly6G (Gr-1 epitope) staining of spleen tissue sections revealed red pulp expansion accompanied by myeloid and megakaryocyte cell accumulation indicating extramedullary hematopoietic activity in the shITGB4/KO group (Fig. 8c). Conversely, the intrasplenic lymph follicles almost disappeared in the shITGB4/KO group according to morphological criteria (Fig. 8c). Similar observations were made with spleens from mice on day 10 after s.c. injection of PaCa5061 cells (Additional file 7: Fig. S7).

In the BM of shITGB4 tumor-bearing WT mice, we found decreased levels of CD11b^+^ Gr-1^+^ cells stemming from the Gr-1^Hi^ SCC^Int^ fraction (see population A in Additional file 6: Fig. S6C) whereas the percentage of Gr-1^Hi^ SCC^Lo^ cells (see population B in Additional file 6: Fig. S6C) was increased in both shITGB4 tumor-bearing WT and E-/P-selectin KO mice (Fig. 8D).

These findings suggested that CD11b^+^ Gr-1^+^ cell trafficking might depend on host E-/ P-selectin expression giving rise to the question of whether these cells are capable of binding E-/ P-selectin. In fact, murine CD11b^+^ Gr-1^+^ leukocytes isolated from s.c. PC-3 ITGB4 KD tumor nodules showed considerable static murine E- and P-selectin binding capacity (Additional file 8: Fig. S8).

### Combined ITGB4 depletion and E-/P-selectin knockout cooperatively impairs tumor growth in fully immunocompetent hosts

**Fig. 9:**
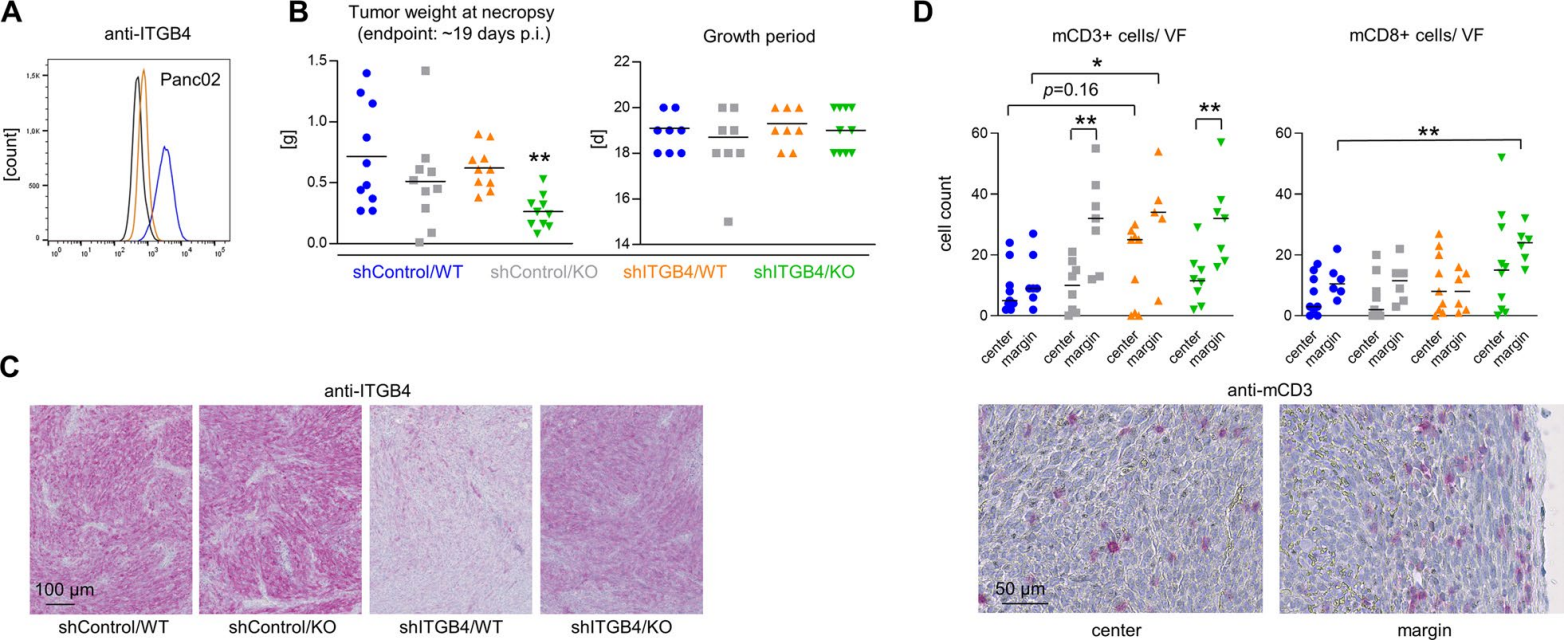
Combined depletion of ITGB4 and E-/P-selectin synergistically reduces pancreatic cancer growth in fully immunocompetent mice. mITGB4 knockdown status in Panc02 murine pancreatic cancer cells prior injection (**A**). Tumor weight at necropsy and growth periods after s. c. injection of Panc02 control vs. ITGB4 knockdown cells into E-/P-selectin wildtype vs. knockout C57BL/6 mice (**B**). Representative anti-mITGB4 immunostaining images of Panc02 tumor tissues (**C**). Quantification of murine CD3^+^ and CD8^+^ lymphocytes per viewing field (VF) in the center and at the margin of s.c. Panc02 tumors across the different groups and representative anti-mCD3 immunostaining images from the center and margin of a Panc02 tumor. Black lines in scatter plots represent mean values. **p < 0.01

Based on our observation that ITGB4 KD significantly alters the local and systemic immune environment during initial tumor formation depending on the E-/P-selectin status of the host, we next investigated whether a cooperatively delayed tumor formation can also be observed after combined depletion of ITGB4 and E-/P-selectin in a syngeneic tumor model using fully immunocompetent hosts. For this purpose, murine pancreatic adenocarcinoma cells Panc02 with or without stable ITGB4 KD (Fig. 9A) were injected into WT or E-/P-selectin KO C57BL/6 mice. Intriguingly, both the E-/P-selectin KO and the ITGB4 KD alone had no significant effect on tumor growth, whereas in the combination group the resulting tumor weights at necropsy were significantly reduced after similar growth periods (Fig. 9B). Stable depletion of ITGB4 KD after in vivo growth of Panc02 cells was validated by IHC (Fig. 9C). Again, the KD efficiency was apparently less striking in the E-/P-selectin KO environment (Fig. 9C). Intriguingly, the ITGB4 status of the TCs and the E-/P-selectin status of the tumor stroma also affected the local immune environment in these immunocompetent hosts: under both conditions per se, the number of CD3^+^ cells specifically increased at the tumor margin, but not in the tumor center (Fig. 9D). Of note, the ITGB4 KD alone was by trend accompanied by a higher number of CD3^+^ cells in the tumor center (*p* = 0.16), which was not detectable upon combined depletion of ITGB4 and E-/P-selectin KO (Fig. 9D). The number of CD8^+^ T cells was significantly increased at the tumor margin only after combined depletion of ITGB4 and E-/P-selectin (Fig. 9D).

### High ITGB4 levels indicate poor outcome in R0-resected serous ovarian cancer patients and negatively correlate with the number of tumor-associated leukocytes

**Fig. 10:**
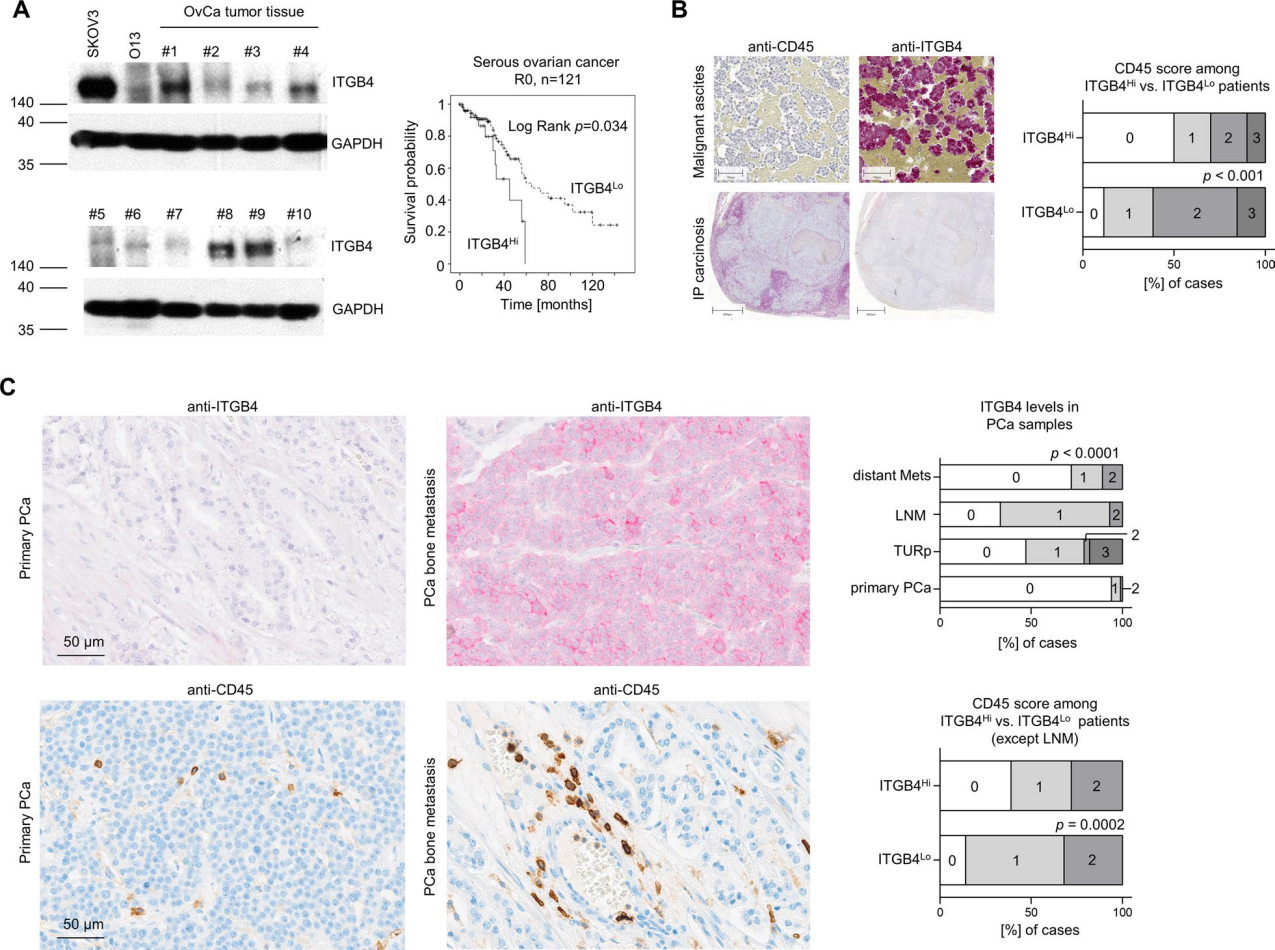
ITGB4 has prognostic relevance and inversely correlates with the number of tumor-associated CD45^+^ leukocytes in patients. Representative ITGB4 and corresponding GAPDH Western blot bands in ovarian cancer (OvCa) tissue samples. Overall survival of ITGB4^Lo^ (75%) vs. ITGB4^Hi^ (25%) R0-resected serous ovarian cancer patients (**A**). Representative anti-CD45 and anti-ITGB4 immunostaining images from consecutive intraperitoneal (IP) carcinosis and malignant ascites ovarian cancer patient samples and calculation of the association between both parameters (**B**). Representative anti-ITGB4 and anti-CD45 immunostaining images from primary and metastatic prostate cancer specimens, ITGB4 levels in different types of prostate cancer samples and association between ITGB4 and number of tumor-associated leukocytes in these samples except lymph node metastases (LNM) (**C**). TURp = palliative transurethral resection

To test the translational relevance of our findings, the prognostic role of ITGB4 and its putative negative correlation with the number of tumor-associated leukocytes was examined in patient material. First, an ovarian cancer patient cohort (n = 252) that contained surgical specimens of primary ovarian cancers was used to study the prognostic role of ITGB4. Patients suffering from local recurrence, borderline, and benign tumors were excluded (n = 219). ITGB4 expression was examined at the protein level from tumor lysates and normalized to GAPDH levels as detected by WB analyses (Fig. 10A). For the classification of low vs. high ITGB4 expression, the cut-off was set according to the 3rd quartile, so that the low ITGB4 expression group included 75% and the high ITGB4 expression group included 25% of all cases. The parameters age, grading, FIGO stage, lymph node involvement, distant metastases, histology, and surgical outcome were used for statistical analysis. Using Pearson's chi-square test, no significant correlations were found with respect to ITGB4 expression in the parameters surveyed. However, because histologic subtype and macroscopically visible residual tumors at surgery are of paramount biologic and prognostic significance in ovarian cancer, respectively, the cohort was further narrowed to cases with serous ovarian cancers and macroscopically tumor-free surgery only (n = 121). In that cohort, the overall survival time estimated according to Kaplan–Meier was significantly prolonged in the low ITGB4 expression group (Log Rank (Mantel-Cox) *p* = 0.034, Fig. 10A).

Second, in a collection of ovarian cancer formalin-fixed, paraffin-embedded (FFPE) tissues including specimens of intraperitoneal carcinosis and malignant ascites (n = 46), we additionally analyzed the level of ITGB4 expression in the TCs and the presence of tumor-associated CD45^+^ leukocytes based on the respective IHC scores ranging from low (0 + 1) to high (2 + 3) (ITGB4) and 0 to 3 (CD45). Intriguingly, we observed a highly significant inverse association of ITGB4 and the level of tumor-associated CD45^+^ leukocytes (*p* < 0.001, Fig. 10B).

Furthermore, while ITGB4 is almost absent in the TCs of primary prostate cancer specimens, the percentage of ITGB4^+^ TCs drastically increases in locally advanced/recurrent tumors (treated with palliative transurethral resection, TURp), lymph node and distant metastases (Fig. 10C). Considering the number of tumor-associated leukocytes based on anti-hCD45 stainings on consecutive slides of primary, locally advanced and distant metastasis samples revealed a highly significant inverse association with the ITGB4 level. Patients with higher ITGB4 expression showed lower levels of immune cell infiltration and vice versa (Fig. 10C). Lymph node metastases were not considered as tumor-associated leukocytes cannot be distinguished from resident leukocytes.

Finally, the TCGA and GTEx databases were used to verify a weak, but significant inverse correlation of CD45 (*PTPRC* gene), CCR5 and CCR1 with ITGB4 in human pancreatic cancer (Additional file 9: Fig. S9A), but not normal pancreas (Additional file 9: Fig. S9B). CCR5 and CCR1 were tested as they are important receptors for some of the chemokine ligands found to be up-regulated in supernatants of PaCa5061 shITGB4 cells.

## Discussion

Based on our hypothesis that the leukocyte adhesion cascade is most important in the process of hematogenous metastasis, ITGB4 emerged as a possible driver of metastasis identified using the relatively E-/P-selectin-independent metastatic prostate cancer model [24]. However, after depletion of ITGB4, the main phenotype was already apparent at the primary tumor level in terms of moderately delayed tumor growth. Most pronounced, however, was the super-additively delayed tumor growth in the presence of concomitant ITGB4 KD and E-/P-selectin double deficiency, whereas E-/P-selectin deficiency alone had no such effect on tumor growth. This observation could be validated using completely independent models, some using different routes of administration (s.c., i.p.) and in different mouse strains (*Pfp*^−/−^/*Rag2*^−/−^ C57BL/6, SCID BALB/c, *Rag2*^−/−^ BALB/c)—including a syngeneic model in fully immunocompetent hosts (C57BL/6). Apparently, E-/P-selectin deficiency in the tumor stroma creates a previously unknown vulnerability of TCs to additional ITGB4 depletion, which was also suggested by increased ITGB4 expression in the xenograft tumors in E-/P-selectin KO mice in several of the tested models. The subsequent aim of this study was to explore the fundamental mechanisms underlying this newly observed and apparently entity-spanning synergism at the primary tumor site.

ITGB4 dimerizes exclusively with integrin α6 (ITGA6) to form one major component of hemidesmosomes for binding to laminin-α3β3γ2 (laminin-5). Loss of this TC-ECM contact physiologically leads to induction of intrinsic apoptosis (anoikis) [30]. Therefore, it was reasonable to assume that the delayed tumor growth in the ITGB4 KD group was at least in part due to enhanced apoptosis. Indeed, we detected an increased percentage of apoptotic TCs in all tested xenograft models belonging to the ITGB4 KD groups. In contrast, the proliferation marker Ki-67 was not altered. At the same time, TCs must have been capable of suppressing anoikis to some extent [30], otherwise no tumors at the inoculation site would have grown out. In fact, in addition to the aforementioned signs of apoptosis, we revealed simultaneous increases in p27 and cyclin D3 expression with RAF/MEK-independent MAPK activation and reduced MAPK:p38MAPK ratio specifically in the ITGB4 KD tumors, a molecular signature that has been reported to indicate anoikis resistance development [31]. Activated p42/44 MAPK signaling generates survival signals that counteract cell death induced by loss of matrix contact [32]. These signaling- and cell cycle-related alterations remain to be validated in the further tested models. In vitro, the ITGB4 KD did show reduced proliferation particularly after low density seeding, suggesting loss of growth-promoting autocrine stimuli upon ITGB4 KD. However, the colony-forming ability, which was consistently reduced in 3D soft agar assays in all models, could only be partially rescued by addition of control cell (but not ITGB4 KD) CM in two of the three models (PaCa5061, SKOV3). Hence, further stimuli likely stemming from the TME might also contribute to the survival of ITGB4 KD TCs in vivo (see below). Similarly inconsistent was the detection of an increased proportion of apoptotic cells after 3D culture in the PC-3 and PaCa5061 model only. Using soft agar assays containing the main ITGB4 ligand laminin V, however, a consistent increase in apoptosis and necrosis could be detected upon ITGB4 KD in all tested models, which was accompanied by reduced viability.

The synergism between ITGB4 KD and selectin KO on tumor growth was observed only in the presence of double deficiency of both E- and P-selectin, but not in the presence of single deficiency of either selectin. One major phenotypic difference between E-/P-selectin single vs. double KO mice is a more strikingly reduced leukodiapedesis in double KO mice [8, 27]. Therefore, although we made our observations in mice lacking an adaptive immune system, we hypothesized that in E-/P-selectin double KO mice, fewer leukocytes (of the innate immune system), which may promote the growth specifically of ITGB4 KD TCs, infiltrate the developing tumors. Innate immune cells are in particular considered to be key players of the TME that critically influence tumor progression and the course of therapeutic interventions [33]. Later, the same synergism between ITGB4 and E-/P-selectin on tumor growth was evident in a syngeneic model in fully immunocompetent hosts. The specificity of the tumor-promoting effect of these tumor-infiltrating innate immune cells on ITGB4 KD tumors was evident as the growth of control tumors was not affected by E-/P-selectin deficiency.

Intriguingly, based on immunostainings for mCD45 cells we revealed an increased initial infiltration of murine leukocytes in ITGB4 KD tumor nodules, which was visible in WT, but not in E-/P-selectin KO mice. CM of ITGB4 KD cells attracted significantly more leukocytes in 3D invasion assays than CM of control cells independent of the tested model. For each model, individual chemokines were detected to be elevated in the supernatants of ITGB4 KD cells, which could be partly reverted by STAT inhibition. More detailed ex vivo flow cytometric analyses of leukocyte subpopulations revealed an increased percentage of CD45^+^ CD11b^+^ Gr-1^Hi^ SSC^Lo^ cells in the initial ITGB4 KD tumor nodules, whereas these changes were no longer evident in E-/P-selectin double KO mice. This marker combination defines immature myeloid cells in mice, widely considered to be myeloid-derived suppressor cells (MDSCs) [34]. MDSCs have been extensively described as negative modulators of immunological and non-immunological processes and have been positively associated with tumor progression and metastasis in several human and murine studies [35–39]. Chemokines are supposed to be key mediators of MDSC recruitment and the chemokines found in supernatants of ITGB4 KD TCs can be largely associated with an attraction of MDSCs (CCL2 [40]; CCL5 [41]; CXCL1/2 [42]; CXCL17 [43], see Fig. 7B). In addition to the secretion of chemokines, an increased number of extracellular vesicles was detected in the ITGB4 KD of two of the models tested. This finding suggests that ITGB4 KD directly or indirectly affects the mechanism of exocytosis. Tumor-derived extracellular vesicles were able to induce differentiation of normal myeloid cells into MDSCs [44]. Further analyses revealed that the enhanced leukocyte infiltrate of ITGB4 KD tumor nodules was characterized by arginase-1- and myeloperoxidase-positive cells. These findings collectively support the hypothesis that in ITGB4 KD tumors, actively recruited MDSC may be critical for tumor growth. Based on our mouse models, such tumor-promoting effect of MDSCs would interestingly be independent of T cells. A direct link between ITGB4 expression and inflammatory responses exists so far only in a mouse model with conditional ITGB4 KO in epithelial cells of the airways. Here, the loss of ITGB4 expression leads to an excessive increase of inflammatory cells in the lung tissue [45], supporting the present observations.

Besides these effects on the local tumor immune environment, the ITGB4 KD also changed the systemic immune environment within only 10 days upon TC inoculation. Thus, the BM of mice with ITGB4 KD tumors also showed an increased percentage of CD11b^+^ Gr-1^Hi^ SSC^Lo^ cells (apparently at the expense of more mature myeloid cells, *i.e*. CD11b^+^ Gr-1^Hi^ SSC^Int^ cells). The same subpopulation was increased in blood and, as already mentioned, tumors in the ITGB4 KD group, provided they were E-/P-selectin-competent animals. In the case of simultaneous E-/P-selectin KO, however, the CD11b^+^ Gr-1^Hi^ SSC^Lo^ cells were no longer increased in the blood and in the tumors, but in the BM. This finding indicates that the ITGB4 KD propagates the expansion of immature myeloid cells in the BM, while their mobilization into the blood and subsequent infiltration of the tumor depends on E-/P-selectin. Furthermore, E-/P-selectin KO mice with ITGB4 KD tumors showed an astonishing disruption of splenic morphology (almost complete loss of follicular structures) accompanied by a decrease in splenic CD11c^+^ cells (presumably dendritic cells, DCs), which were concomitantly increased in the tumor nodules. Importantly, these observations were not made with E-/P-selectin wildtype mice with ITGB4 KD tumors nor with E-/P-selectin KO mice bearing control tumors. Whether CD11c^+^ cells are recruited from the spleen into the tumors (in the shITGB4/KO group) and whether this leukocyte trafficking is responsible for the over-additively reduced tumor growth and/or lack of splenic follicular structures remains to be determined by future experiments.

Importantly, elevated ITGB4 levels were associated with a worse prognosis of R0-resected patients with serous ovarian cancer, supporting a recent study identifying ITGB4 as an independent predictor of adverse progression-free survival in ovarian cancer [46]. Moreover, the ITGB4 level was inversely associated with the presence of tumor-associated CD45^+^ leukocytes. More specific immunohistochemistry of human MDSCs in clinical material was not initially attempted because, unlike murine MDSCs, human MDSCs have a more complex phenotype and can only be identified using multi-color flow cytometry and not FFPE tissues [47]. As possible markers for phenotyping of human MDSCs the antigens CD11b, CD14, CD15, CD66, and HLA-DR could be used [34]. Database analyses also revealed a significant negative correlation between ITGB4 and PTPRC (CD45 gene), CCR5 as well as CCR1 in pancreatic cancer, but not normal pancreas tissue. ITGB4 has been proposed as a potential biomarker for pancreatic adenocarcinoma due to its overexpression and altered localization compared to normal and chronically inflamed pancreatic tissues [48]. In pancreatic cancer, increased ITGB4 expression implies enhanced invasiveness [49], is associated with molecular features indicative of epithelial-mesenchymal transition (EMT), and independently predicts poor outcome [50]. For prostate cancer, controversial data are available. While earlier studies reported reduced expression of ITGB4 in both prostatic intraepithelial neoplasms and invasive prostate carcinomas [51], later publications are available showing overexpression of ITGB4 in more aggressive prostate carcinomas [52]. In our analyses of clinical specimens, we observed a clear induction of ITGB4 during prostate cancer progression. Moreover, we could again confirm the inverse association of the ITGB4 level and number of tumor-associated leukocytes in prostate cancer samples.

## Conclusion

This study shows that depletion of ITGB4 in TCs of several tumor entities in different model systems leads to a delay in tumor growth. At the same time, the ITGB4 KD increases the release of various chemokines—apparently via enhanced STAT signaling—resulting in alterations of the local and systemic immune environment. Increased attraction of CD11b^+^ Gr-1^Hi^ SSC^Lo^ cells (*e.g*., MDSCs) appears to be of particular importance for the establishment of ITGB4 KD xenografts and requires endothelial selectins of tumor vessels for infiltration. In the absence of E-/ P-selectins, intratumoral MDSC numbers are reduced and growth of ITGB4-depleted xenografts is overadditively diminished. Clinical material also shows an inverse correlation between ITGB4 expression and number of tumor-associated leukocytes. Our data suggest on the one hand that the unfavorable prognostic effect of high ITGB4 levels might be due to reduced counts of anti-tumor MDSCs. On the other hand, however, our findings suggest that ITGB4^Lo^ patients could specifically benefit from MDSC targeting. These findings emphasize the functional role of molecules of the leukocyte adhesion cascade which regulate the influx of immune-competent cells into tumor nodules. These observations should be considered in the further development of tumor immunotherapies.

## Materials and methods

### Cell culture

Human prostate cancer PC-3 cells, obtained from Deutsche Sammlung von Mikroorganismen und Zellkulturen (DSMZ, Braunschweig, Germany), were cultured in RPMI-1640 (Gibco, Paisley, UK). The human pancreatic adenocarcinoma cell line PaCa5061 was established from a primary tumor and cultured as previously described [53]. Human ovarian cancer cells SKOV3 were purchased from the American Type Culture Collection (ATCC, No. HTB-77) and maintained in McCoy’s 5A medium with 2 mM L-glutamine. The murine pancreatic ductal adenocarcinoma cell line Panc02 was obtained from Division of Cancer Treatment and Diagnosis (DCTD) Tumor Repository (National Cancer Institute, Frederick, MD, USA) and cultured in RPMI-1640. All cell culture media were supplemented with 10% fetal bovine serum, 50 U/mL penicillin and 50 µg/mL streptomycin (all from Gibco). All tumor cell lines were cultivated under standard conditions unless stated otherwise (37 °C, H_2_O-saturated atmosphere, 5% CO_2_). To generate CM, TCs were grown to confluence under standard conditions, washed with PBS and further cultivated under serum-free conditions for 24 h. Afterwards, supernatants were collected, centrifuged at 1500 rpm for 5 min and frozen at -80 °C.

### Generation of ITGB4 knockdown cell lines

Stable ITGB4 KD and control transfectants were established from PC-3, PaCa5061, SKOV3 and Panc02 cell lines by lentiviral transduction. In short, sequences coding for shRNA sequences targeting human ITGB4 (shITGB4: 5’-CGAGAAGCTTCACACCTAT-3’) or firefly luciferase (shControl: 5’-GTGCGTTGCTAGTACCAAC-3’) were cloned using the pLVX-shRNA1 vector (Clontech, Saint-Germain-en-laye, France). Stable, single-cell-derived ITGB4 KD clones were established by puromycin selection, flow cytometric sorting (FACSAria, BD, Heidelberg, Germany) and limiting dilution cloning. To avoid clonal effects, clones with the strongest depletion were pooled. For the murine cell line Panc02, murine ITGB4 KD sequences (m-shITGB4: 5’-GTGGAGAGCAGCCTTGAAAT-3’) and scrambled sequences (m-shControl: 5’-CCTAAGGTTAAGTCGCCCT-3’) were cloned in a pLVX-shRNA1 vector with the puromycin resistance cassette cut out by restriction enzymes AgeI and XhoI (New England Biolabs, Ipswich, MA, USA). After blunting the cohesive ends by a fill-in reaction with Klenow-enyzme (Sigma, St. Louis, MO, USA), the ends were ligated by T4 DNA ligase (New England Biolabs). The Panc02-shITGB4 cells were enriched by ITGB4 negative selection using magnetic MACS cell separation LD columns with the QuadroMACS Separator according to manufacturer specifications (Miltenyi Biotec, Bergisch Gladbach, Germany) after cell labeling with murine REAfinity anti-CD104 (Clone REA456-APC) and anti-APC MicroBeads (Miltenyi).

### Tumor Cell Proliferation Assay

To analyze TC proliferation under sub-confluent two-dimensional conditions, 5 × 10^4^ TCs were seeded into T25-flasks and the resulting cell number was manually counted in a Neubauer counting chamber after six days. The experiment was run in biological triplicates.

### XTT assay

To determine the appropriate cell number for the XTT assay, a growth curve was established for each cell line by diluting the cells in a 96-well plate. The cell number required to reach exponential growth 48 h after seeding was determined (PC-3: 2 × 10^4^ cells, PaCa5061: 3 × 10^4^ cells, SKOV3: 1 × 10^3^ cells). TCs were plated in the pre-determined density onto a 96-well plate in 100 µL medium and incubated for 48 h. As a background control, wells were loaded with medium without cells. According to the manufacturer's instructions (Cell proliferation Kit II (XTT), Roche, Mannheim, Germany), XTT solution was prepared at a ratio of 1:50 and 50 µL per well were added to the cells. After incubation for 4 h under culture conditions, optical density was measured at 490 nm (reference measurement at 630 nm) using an ELISA reader (DIAS MAX 002, Dynex Technologies, Chantilly, USA).

### Soft agar colony formation assay

To allow colony formation within a soft agar matrix, 24-well plates were initially pre-coated by adding 250 µL standard medium or standard medium mixed 1:2 with CM, in both cases containing 0.8% 2-Hydroxyethylagarose (Sigma, St.Louis, MO, USA). After solidification of the bottom layer, 150 TCs diluted in 250 µL of the respective medium/ CM mixture with 0.3% soft agar were seeded into each well and stored for 20 min at 4 °C for solidification. 24-well plates were then incubated under standard conditions. After three days, 1 mL suitable medium or shControl-/shITGB4-CM was added and refreshed during the following incubation period as appropriate. Colony diameters were microscopically evaluated after eleven days (AxioVs40 V 4.7.2.0, Zeiss).

In an additional experimental setup, tumor cell apoptotic and necrotic behavior was examined on a white 96-well plate (Thermo Fisher Scientific #165,306) with and without the addition of laminin V (Biolamina, #LN332-0502). For this purpose, each well was pre-coated with 50 µL of 2% soft agar diluted 1:2 with complete medium and incubated for 30 min at 4 °C to solidify. For each well, 1 × 10^4^ cells were embedded in 50 µL 0.5% soft agar with or without the addition of 10 µg/mL laminin V and also allowed to solidify. RealTime-Glo Annexin V Apoptosis and Necrosis Assay (Promega, #JA1011) was then added according to the manufacturer's instructions and apoptosis and necrosis were measured by using a microtiter plate reader (TECAN GENios, Tecan Trading AG, Switzerland) after 48 h of incubation at 37 °C. To determine the viability of the cells after 72 h, 100 µL Cell Titer Glo 3D (Promega, #G9682)) was added to each well and the luminescence signal was also measured by using the microtiter plate reader.

### Cultivation on PolyHEMA-coated surface and apoptosis assay

Poly-2-hydroxyethyl methacrylate (PolyHEMA) was purchased from Sigma (St.Louis, MO, USA) and prepared by dissolving to 12 mg/mL PolyHEMA in 95% absolute ethanol. T75 culture flasks were covered with 4 mL PolyHEMA solution and incubated at 37 °C in dry atmosphere until the liquid part was evaporated. Afterwards, 1 × 10^6^ TCs per flask were seeded in 10 mL of the respective medium. After three days, flow cytometric analysis of apoptotic TCs was carried out using the Annexin V-FITC Kit (Miltenyi Biotec) according to the manufacturer's instructions. To obtain a single-cell suspension, tumor spheroids were gently digested for 20 min at 37 °C in trypsin–EDTA.

### Human macrophage 3D invasion assay

The potency of TC CM to attract immune cells was investigated by spherical infiltration of human macrophages in a 3D culture system. Primary human macrophages were generated as previously described [54] and embedded into an inner matrix of rat tail collagen I (Corning), which was surrounded by an outer collagen-containing matrix of lower density containing the control medium or CM from the TC cultures (see Fig. 7A for illustration). For the preparation of the inner matrix, 1 × 10^6^ human macrophages were mixed with collagen I solution (1.5 mg/mL) in a ratio of 1:1 and 100 µL each were applied to a flat bottom 96-well plate. Polymerization was carried out within 60 min at 37 °C. In parallel, a 24-well plate was pre-coated with 200 µL collagen I solution diluted 1:1 with macrophage culture medium. Subsequently, the macrophage-collagen matrix was detached from the 96-well plate and centered on each well of the coated 24-well plate. For the outer matrix, CM or control medium were mixed 3:1 with collagen I (1.0 mg/mL) containing M-CSF (10 ng/mL) and added onto the macrophage-collagen matrix. M-CSF was added to improve read out clarity. To protect from desiccation, the prepared wells were mixed with 400 µL of macrophage culture medium and incubated at 37 °C. After 4 h, four microscopic Sects. (10 × magnification) of the invasion front per well were photographed uniformly and the number of invaded macrophages was quantified using ImageJ software (version 2006.02.01). A total of 16 images from two different donors were analyzed per cell line. Each of the cell culture supernatants were measured in four technical replicates.

### Quantification of chemokine concentrations in cell culture supernatants

All ELISA kits were based on the sandwich ELISA method. TCs were washed with PBS when reached 70–80% confluence and covered with serum-free media for 24 h. Cell culture supernatants were harvested, centrifuged to avoid cell debris in the sample, transferred to liquid nitrogen, and stored at -80 °C. Thawed samples were analyzed to quantify chemokine secretion using ELISA kits from R&D Systems (CCL2, CCL5, CXCL1, CXCL2, CXCL10, CXCL17) according to the manufacturer’s instructions. To examine chemokine secretion from TCs after STAT inhibition, fludarabine or nifuroxazide (Sigma) were added to serum-free media at 50 µM (24 h) [55, 56]. As control, the serum-free media was supplemented with 0.1% DMSO.

### Nanoparticle tracking analysis

The concentration and size of microvesicles was determined by nanoparticle tracking analysis (NTA), using an LM14 instrument (NanoSight, Malvern Panalytical) equipped with a 638 nm laser and a Merlin F-033B IRF camera (Adept Electronic Solutions) as previously described. Briefly, microvesicle-enriched samples were diluted 1:300 in PBS prior to NTA. Quadruple one-minute movies were recorded on camera level 15, and then analyzed with detection threshold 4 in NTA 3.2 Build 16. All NTA EV size data is presented as mode values.

### Mouse experiments

Different mouse strains and TC transplantation sites were used according to the tumor entity. For the prostate cancer PC-3 and pancreatic cancer PaCa5061 models *Pfp*^−/−^/*Rag2*^−/−^ C57BL/6 mice with or without E- and P-selectin deficiency were injected s.c. with 1 × 10^6^ shControl or shITGB4 cells [19]. For the ovarian cancer SKOV3 model 1 × 10^6^ shControl or shITGB4 cells were injected i.p. into SCID [21] or *Rag2*^−/−^ Balb/c mice with or without E- and P-selectin deficiency (C.129S6(B6)-*Rag2*^*tm1Fwa*^ N12 or C.129S6(B6)-*Rag2*^*tm1Fwa*^ N12 back crossed with C.129S2(B6)-*Sele*^*tm1Hyn*^* Selp*^*tm1Hyn*^/J)). The endpoint of these experiments was the development of a s.c. xenograft tumor of 1–1.5 cm^3^ (PC-3, PaCa5061) or the generation of palpable abdominal tumor masses or ascites (SKOV3). With these models, the effect of the ITGB4 KD and E-/P-selectin KO on tumor development and hence overall survival was analyzed. To determine the degree of intraperitoneal (IP) carcinosis in the SKOV3 model, an IP carcinosis score (ICS) was determined at necropsy as illustrated in Fig. 3B. Malignant cells were harvested from ascites samples by flushing the abdominal cavity with 5 mL PBS, then aspirating all intraabdominal fluid (PBS + ascites) and collecting it in a centrifugation tube. After centrifugation, cells were fixed in 4% formalin for 20 min, washed twice with PBS, and immobilized in 2% agar NOBLE (Difco) before paraffin embedding.

To study the role of ITGB4 and E-/P-selectin on extravasation independently of primary tumor growth, 1 × 10^5^ shControl or shITGB4 PC-3 cells in 100 µL medium without supplements were injected i.v. into *Pfp*^−/−^/*Rag2*^−/−^ mice with or without E-/P-selectin deficiency (n = 4–7). These mice were sacrificed and analyzed for the presence of macro-metastases eight weeks after injection.

To explore whether double or single deficiency of E- and/or P-selectin are required to achieve synergistic tumor growth delay together with the ITGB4 KD, 1 × 10^6^ shControl or shITGB4 PC-3 cells were s.c. injected into *Pfp*^−/−^/*Rag2*^−/−^ mice with or without E-selectin (*Sele*^−/−^), P-selectin (*Selp*^−/−^) or E-/P-selectin (*Sele*^−/−^/*Selp*^−/−^) KO (in 200 µL medium without supplements). The endpoint of this experiment was the development of s.c. primary tumors of approximately 1 cm^3^.

To investigate the level of leukocyte infiltration into developing xenograft tumors, 1 × 10^6^ shControl or shITGB4 PC-3 or PaCa5061 cells were s.c. injected in 200 µL medium without supplements into *Pfp*^−/−^/*Rag2*^−/−^ mice with or without E-/P-selectin deficiency. Mice were sacrificed four days after injection and initial tumor nodules at the injection site were harvested for subsequent IHC analysis.

To investigate the local and systemic immune cell composition during the early tumor growth phase, 1 × 10^6^ shControl or shITGB4 PC-3 cells were s.c. injected in 200 µL medium without supplements into *Pfp*^−/−^/*Rag2*^−/−^ mice with or without E-/P-selectin deficiency. On day ten after injection, mice were finally anesthetized, blood samples were taken by cardiocentesis and mice were sacrificed by cervical dislocation. S.c. tumor nodules and spleens were excised. BM was harvested by flushing tibia and femur of one hindlimb with 1 mL NaCl. All tissues/organs were prepared for subsequent flow cytometric analysis. For this purpose, tumor nodules were cut into pieces and enzymatically digested in 1 mg/mL DNase I (Roche Diagnostics), 2.4 U/mL dispase (Gibco), 1 mg/mL hyaluronidase (Sigma-Aldrich) and 60 µg/mL collagenase (Sigma-Aldrich) for 60 min at 37 °C. Spleens were dissociated into single-cell suspension by mechanical disruption mashing the tissues through 40 µm cell strainers. Erythrocytes of peripheral blood, BM and spleen suspensions were lysed using red blood cell (RBC) lysis buffer (Biolegend). 1 × 10^6^/100 µL cells were subjected to multicolor staining as described below.

Based on our observation that the initial s.c. tumor growth period over ten days leads to a significant decrease in the number of intrasplenic CD11c^+^ cells, additional *Pfp*^−/−^/*Rag2*^−/−^ mice with or without E-/P-selectin deficiency were s.c. injected with 1 × 10^6^ shControl or shITGB4 PC-3 or PaCa5061 cells and spleens were harvested on day ten for FFPE histology/IHC.

To study whether the observed synergism is also present in fully immunocompetent hosts, 1 × 10^6^ shControl or shITGB4 Panc02 cells were s.c. injected in 200 µL medium without supplements into C57BL/6 mice with or without E- and P-selectin deficiency (RRID:IMSR_JAX:008,437 and littermate controls). The resulting tumor weights were analyzed ~ day 19 post injection.

All described animal experiments were permitted by the local animal welfare committee (Project No’s.: N125/2020, G08/75, G15/19, G09/58, G 09/88, G15/102).

### DNA isolation and quantification of disseminated and circulating tumor cells by *Alu*-qPCR

Disseminated TCs were quantified within the blood and left lungs of xeno-engrafted mice at necropsy using human-specific *Alu*-qPCR (the right lungs were fixed in formalin and embedded in paraffin for subsequent histology). DNA was isolated and concentrations determined as previously described [24]. For blood and lung samples DNA concentrations were normalized to 10 ng/µL and 30 ng/µL, respectively, using AE buffer from the isolation kit, and 2 µL per sample were used as template for the SYBR Green-based qPCR in a LightCycler 480 (Roche, Basel, Switzerland). The tissue-specific standard curves and human-specific *Alu* DNA primers were prepared and used as previously described [25]. The measured values of the *Alu*-qPCR analyses were expressed as the number of human cells per total amount of DNA used.

### Flow cytometry

For flow cytometry, human TCs were trypsinized, washed with PBS and stained with anti-CD104 (eBioscience; clone 439-9B) or rat IgG2b (eBioscience eB149/10H5) at a final concentration of 1 µg/mL at 4 °C for 15 min. For the murine cell line Panc02, REAffinity anti-CD104 (Miltenyi; Clone REA456, APC) or REA Control human IgG1 REAffinity (Miltenyi; Clone REA293, APC) were used. The flow cytometric analysis of human TCs from in vitro culture was executed with the CyFlow® Cube 8 cytometer (Partec, Görlitz, Germany) and analyzed with the DeNovo software FCS Express 4.0. In case of the apoptosis assay and detection of ITGB4 levels on Panc02 cells, the MACSQuant® Analyzer 10 flow cytometer (Miltenyi, Bergisch Gladbach, Germany) was used and data were analyzed with the Flowlogic™ software (Inivai Technologies, Mentone VIC, Australia).

For immunophenotyping of single-cell suspensions generated from s.c. xenograft tumors, murine BM, blood and spleen, viable cells were first distinguished with the Zombie Green Fixable Viability Kit (Biolegend). Afterwards, Fc receptors were blocked with anti‐CD16/CD32 mAb (1:50 dilution) and Human TruStain FcX (1:20 dilution) for 10 min at 4 °C. Cells were then stained with fluorophore-conjugated anti-CD11b (clone M1/70.15 from Invitrogen), anti-CD45 (clone 104), anti-CD11c (clone N418), anti-F4/80 (clone BM8) and anti-Gr-1 (clone RB6-8C5) antibodies obtained from Biolegend for 15 min at 4 °C. Following two washing steps with FACS buffer, cells were fixed with Cytofix Fixation solution (BD Bioscience). Stained cells were analyzed on a LSR Fortessa Cell Analyzer (BD Biosciences) (FACS Core Facility, UKE) and data evaluation was carried out with FlowJo v10 software. CD11b^+^ Gr-1^+^ cells were isolated from s.c. PC-3 ITGB4 KD tumors grown in *Pfp*^−/−^/ *Rag2*^−/−^ mice using the Dead Cell Removal Kit (Miltenyi, 130–090-101) first and the MDSC isolation kit afterwards (Miltenyi, #130–094-538) according to the manufacturer’s instructions. Isolated leukocytes were immediately characterized for static murine E- and P-selectin binding capacity in a flow cytometric approach as previously described [18].

For ex vivo quantification of the percentage of ITGB4^+^ xenograft TCs, freshly harvested tumors were cut into millimeter-thin pieces with a scalpel in cell culture medium, passed through a cell strainer (100 µm) using a syringe plunger, washed, and stained at 4 °C with anti-CD104-eFluor660. After 10 min of incubation, samples were transferred to FACS tubes, stained with propidium iodide and analyzed in the CyFlow® Cube 8 cytometer.

### Western blot

The fresh frozen xenograft tumor samples were ground in liquid nitrogen with a mortar and pestle and homogenized with the addition of protein lysis buffer followed by protein extraction, quantification, SDS-PAGE and blotting as described before [57]. Afterwards, the following specific primary antibodies were used: HSC70 (B-6), Neu (ER23), p27 (F-8) (all from Santa Cruz Biotechnology, CA, USA); Cyclin D1 (92G2), Cyclin D3 (DCS22), pan-Akt (40D4), p-Akt (S473) (D9E), p-FAK (Tyr925), pGSK-3β (Ser9), p-JNK (Thr183/Tyr185) (81E11), p-MEK 1/2 (Ser217/221), pmTOR (Ser2481), p-Raf (S338), p-Src Family (Tyr416) (D49D4), pS6 (Ser235/236), p38MAPK (D13E1), p42/44 MAPK (137F5), p-p42/44 MAPK (Thr202/Tyr204) (D13.14.4E) (all from Cell Signaling Technology, MA, USA). Protein levels were quantified using the LAS-3000 Imager and AIDA Image Analyzer v.3.44 from Fuji software (Raytest, Straubenhardt, Germany).

Western Blots of the ovarian cancer patient collective were performed as described previously [58]. For detection, primary antibodies against ITGB4 (clone H1) and GAPDH (clone FL-335) (all from Santa Cruz Biotechnology) were used.

### Immunohistochemistry

IHC staining was performed on FFPE tissue sections. Sections were deparaffinized in descending ethanol concentrations and pre-treated as listed in Additional file 10: Supplementary Table 1. After incubation for 1 h at room temperature with the corresponding primary antibodies (Additional file 10: Supplementary Table 1), samples were washed twice with TBS-T (TBS + 0.1% TWEEN-20) and once with TBS for 5 min. After incubation with the corresponding secondary antibody (Additional file 10: Supplementary Table 1) for 30 min at room temperature, antibody binding was visualized using the Vectastain ABC-AP Kit (VectorLabs., Burlingame, CA, USA) and Permanent Red Solution (Dako) according to the manufacturer’s instruction. In some cases, nuclei were counterstained in Mayer’s hemalum solution. For the hCD45 staining, mouse monoclonal anti-CD45 (LCA) (2B11 & PD7/26) was used in a Ventana BenchMark immunostainer (Roche, Mannheim, Germany).

### Integrin qPCR profiler array

For screening integrin expression levels among human prostate cancer cells with divergent metastatic potential, the RT^2^ Profiler PCR Array for human extracellular matrix/ cell adhesion molecules (PAHS-013ZF, SABiosciences, Qiagen) was applied in accordance with the manufacturer’s instructions. For the PCR reaction a Light Cycler® 480 II (Roche) was used and data were analyzed using the QIAGEN online tool.

### Patient material

252 samples of ovarian cancer patients surgically treated at the University Medical Center Hamburg-Eppendorf between 1996 and 2014 were included to study the effect of ITGB4 expression on overall survival. Patients underwent surgical debulking according to current German guidelines. All patients gave written consent for the use of their tissue and clinical records according to the Investigation Committee and Ethics Committee guidelines (#200,814). Clinical data such as histologic classification or treatment were obtained from detailed databases. The clinical course of all patients was followed from the time of initial diagnosis until the end of 2015. Tissue samples were obtained during surgery and frozen directly in liquid nitrogen. When necessary, surrounding tissue was removed from the tissue samples so that approximately 100 mg of tissue containing at least 70% TCs could be used for protein extraction.

145 primary prostate cancer samples and 62 distant metastases of prostate cancer were stained for ITGB4 and CD45. The primary prostate cancer cohort included patients who were treated in the Hospital of Goeppingen, Germany, between 2002 and 2014. The metastases were derived from patients who were treated at the University Hospital Schleswig–Holstein, Campus Luebeck, Germany, between 1996 and 2015. The Internal Ethical Review Board of the University Hospital Schleswig–Holstein, Campus Luebeck approved the present study. IHC was performed on formalin-fixed, paraffin-embedded tissue collected on tissue microarrays (TMA). Tissue blocks were cut into 6 μm thin sections, mounted on slides, stained by hematoxylin/eosin and relevant tissue regions were circled. Three representative cores of the circled region measuring 0.6 mm in diameter from each sample were assembled into tissue microarray donor blocks using a semiautomatic tissue arrayer.

ITGB4 expression on TCs was categorized into negative (0), weak (1), intermediate (2) and high (3) per visual evaluation. There was no significant heterogeneity between the three tumor spots. Infiltration with CD45^+^ leukocytes was quantified per visual evaluation and categorized into minor infiltration (0–1% = 0), low infiltration (1–10% = 1) and dense infiltration (> 10% = 2). For calculating the association between ITGB4 and CD45, the ITGB4 levels were dichotomized into low (0 + 1) vs. high (2 + 3).

The Gene Expression Profiling Interactive Analysis 2 (GEPIA2) online tool (http://gepia2.cancer-pku.cn) was used to further verify the correlation of genes of interest in the TCGA pancreatic cancer cohort (Pearson correlation analysis). The same analyses were carried out with gene expression data from normal pancreas samples from the Gentoype-Tissue Expression (GTEx) portal.

### Statistics

Data with repeated measurements per sample (several viewing fields) were analyzed using mixed effects models with a random effect for the sample accounting for between-sample variability and dependencies within samples. The model further contained fixed effects for the ITGB4 KD condition and cell line to obtain a cell line-adjusted effect of the condition.

Data with several wells from the same donor each containing repeated measurements for different viewing fields were also analyzed using mixed effects models. Here the random effect was modeled as nested, *e.g.* well nested in donor. These models also further contained fixed effects for condition and cell line to obtain cell line-adjusted condition effects.

Effects for linear mixed effects models were estimated using R version 3.5.3 (R Core Team (2019). R: A language and environment for statistical computing. R Foundation for Statistical Computing, Vienna, Austria; https://www.R-project.org/) and R-packages “lme4” and “lmerTest”. Effects are presented as estimated coefficient with 95% confidence intervals together with corresponding p-values based on Satterthwaite's degrees of freedom method.

Time-to-event data were analyzed using Cox proportional hazards regression models which included cell line and condition as predictors to obtain cell line-adjusted effects of the condition. Assumptions were checked visually. Models were estimated using R version 3.5.3 applying the R-package “survival” (Therneau T. A package for survival analysis in R. R package version 3.1–12; 2020).

## Supplementary Information

**Additional file 1. Suppl. Fig. S1:** Incidence of macrometastases and pulmonary metastatic cell loads at necropsy eight weeks after tail vein injection of PC-3 cells. Bar charts represent mean+SD. *p<0.05; **p<0.01

**Additional file 2. Suppl. Fig. S2:** Cox proportional hazards regression model including cell line and KD condition as predictors to obtain cell line-adjusted effects of the ITGB4 knockdown condition on mouse survival (experiments shown in Figs. 1D, Fig. 2A and 3A).

**Additional file 3. Suppl. Fig. S3:** Western Blot analyses of PC-3 xenograft tumor samples. Exemplary Western Blot images and corresponding quantification of cell cycle- and survival-related protein levels relative to HSC70. Proteins were extracted from PC-3 xenograft tumors from the experiment shown in Fig. 1D. The combination group (shITGB4/KO) is missing since in this group only one mouse developed a xenograft tumor. Bar charts represent mean+SD of n=5. *p<0.05; **p<0.01.

**Additional file 4. Suppl. Fig. S4:** Enhanced attraction of tumor-infiltrating leukocytes in ITGB4-depleted xenografts. Morphology of control and ITGB4 KD tumors in WT and KO mice based on HE stainings (A). Arginase-1, myeloperoxidase (MPO) and inducible nitric oxide synthase (iNOS) expression in control vs. ITGB4 KD PC-3 tumor nodules on d8 after engraftment (B). Intraperitoneal (IP) carcinosis score (ICS) on day 49 after injection of SKOV3 control vs. ITGB4 knockdown cells into E-/P-selectin wildtype vs. knockout rag2-/- BALB/c mice. Representative anti-mCD45 immunostaining images of formalin-fixed, paraffin-embedded intraperitoneal lavage (C). Black lines in the scatter plot represent mean values. **p<0.01; ***p<0.001.

**Additional file 5. Suppl. Fig. S5:** Attraction of human macrophages by ITGB4 knockdown tumor cell-conditioned media. Entire dataset of data shown in Fig. 7A including further donors.

**Additional file 6 Suppl. Fig. S6:** Representative density plots of ex vivo flow cytometric analyses of blood (A), spleen (B) and bone marrow (C) samples of s.c. tumor nodule-bearing mice (10 days after injection of PC-3 control vs. ITGB4 knockdown cells into E-/P-selectin wildtype vs. knockout mice, see Fig. 8 for differences between the groups). Note the populations assigned A-D in SSC-A/ Gr-1 plots (referring to Fig. 8).

**Additional file 7. Suppl. Fig. S7:** Validation of alterations in the spleen morphology ten days after s.c. injection of tumor cells. Representative photomicrographs of HE- and anti-Ly6G (Gr-1 epitope)-stained spleen samples on d10 after s. c. injection of PaCa5061 control vs. ITGB4 knockdown cells into E-/P-selectin wildtype vs. knockout mice. The arrows indicate megakaryocytes, arrowheads indicate immature granulocytes with ring-shaped nuclei.

**Additional file 8. Suppl. Fig. S8:** Static murine E- and P-selectin binding capacity of ITGB4 KD tumor-derived MDSCs. Flow cytometric analysis of CD11b+ Gr-1+ leukocytes isolated from s.c. PC-3 ITGB4 KD tumors regarding static binding of murine E- and Pselectin as indicated. Grey lines in histograms indicate unstained controls.

**Additional file 9. Suppl. Fig. S9:** Validation of inverse correlation of ITGB4 and leukocyte markers in pancreatic cancer. Correlation of ITGB4 and CD45 (PTPRC gene), CCR5, or CCR1 in the human pancreatic ductal adenocarcinoma (PDAC) database of the cancer genome atlas (TCGA) (A) and normal human pancreas expression data of the Genotype-Tissue Expression (GTEx) portal (B) as determined by using the Gene Expression Profiling Interactive Analysis 2 (GEPIA2) online tool (http://gepia2.cancer-pku.cn). TPM = transcripts per million. R- and p-values were calculated based on Pearson correlation analysis.

**Additional file 10. Suppl. Table 1:** Short IHC protocols relevant to the study (IHC-P).

## Data Availability

The datasets generated and/or analyzed during the current study are available in the Gene Expression Profiling Interactive Analysis 2 (GEPIA2) online tool (http://gepia2.cancer-pku.cn).

## Abbreviations

*Alu*-PCR: Quantitative realtime-PCR for human *Alu* sequences
APC: Allophycocyanin
BM: Bone marrow
CM: Conditioned medium
CTCs: Circulating tumor cells
ECM: Extracellular matrix
ECs: Endothelial cells
ELISA: Enzyme-linked immunosorbent assay
FC: Flow cytometry
FFPE: Formalin-fixed, paraffin-embedded
GTEx: Genotype-tissue expression
HE: Hematoxylin–eosin
Hi: High
ICS: Intraperitoneal carcinosis score
IHC: Immunohistochemistry
Int: Intermediate
i. p.: Intraperitoneal
ITGB4: Integrin subunit β4
i. v.: Intravenous
KD: Knockdown
KO: Knockout
LNM: Lymph node metastases
LN V: Laminin-V (laminin-332)
Lo: Low
MDSCs: Myeloid-derived suppressor cells
Mets: Metastases
OvCa: Ovarian cancer
PCa: Prostate cancer
PDAC: Pancreatic ductal adenocarcinoma
PI: Propidium iodide
Poly-HEMA: Poly-2-hydroxyethyl methacrylate
s. c.: Subcutaneous
SCID: Severe combined immunodeficiency
shRNA: Short hairpin RNA
SSC: Side scatter
TCGA: The Cancer Genome Atlas
TCs: Tumor cells
TME: Tumor microenvironment
TPM: Transcripts per million
TURp: Palliative transurethral resection
VF: Viewing field
WB: Western blot
WT: Wildtype
XTT: 2,3-Bis-(2-Methoxy-4-Nitro-5-Sulfophenyl)-2*H*-Tetrazolium-5-Carboxanilid
