# Supplementary figures and images for: Tumor cell integrin β4 and tumor stroma E-/P-selectin cooperatively regulate tumor growth in vivo
Source: J Hematol Oncol. 2023 Mar 17;16:23. doi: 10.1186/s13045-023-01413-9 (PMC10022201; doi:10.1186/s13045-023-01413-9)

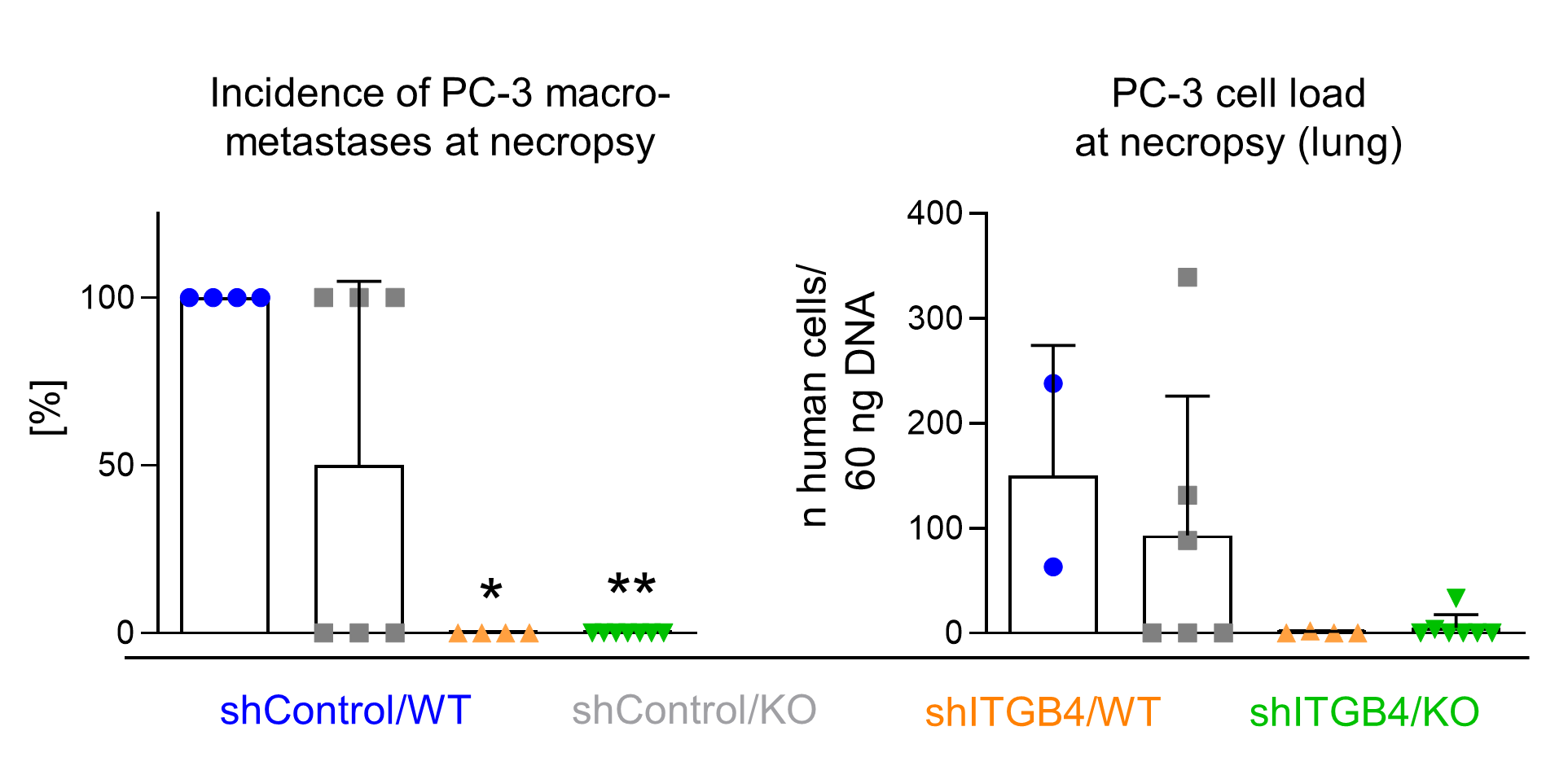

Supplement: Supplementary file 1 — Additional file 1. Suppl. Fig. S1: Incidence of macrometastases and pulmonary metastatic cell loads at necropsy eight weeks after tail vein injection of PC-3 cells. Bar charts represent mean+SD. *p<0.05; **p<0.01 [file 13045_2023_1413_MOESM1_ESM.tif]

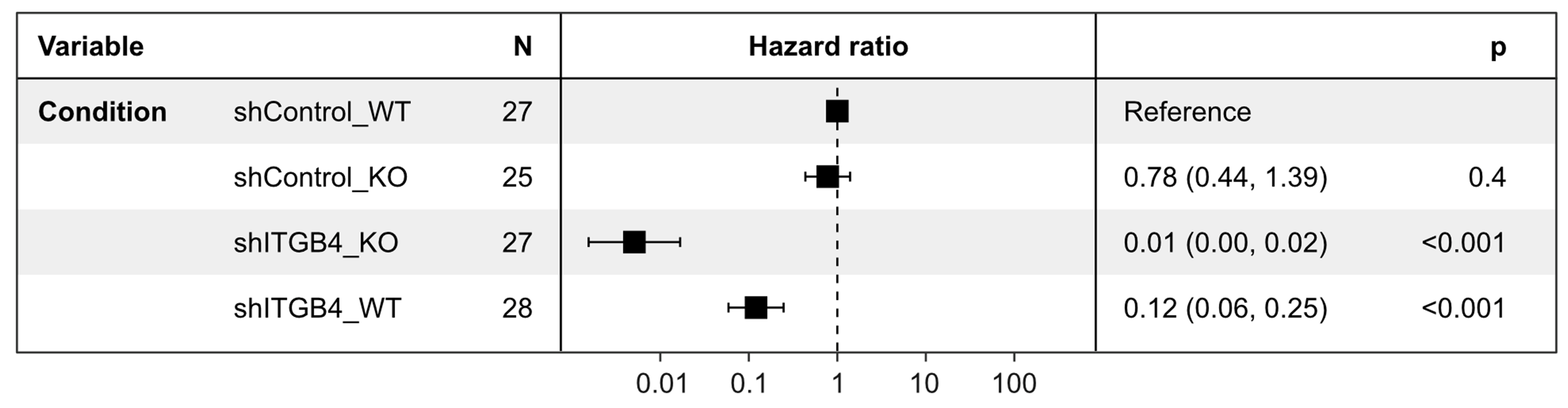

Supplement: Supplementary file 2 — Additional file 2. Suppl. Fig. S2: Cox proportional hazards regression model including cell line and KD condition as predictors to obtain cell line-adjusted effects of the ITGB4 knockdown condition on mouse survival (experiments shown in Figs. 1D, Fig. 2A and 3A). [file 13045_2023_1413_MOESM2_ESM.tif]

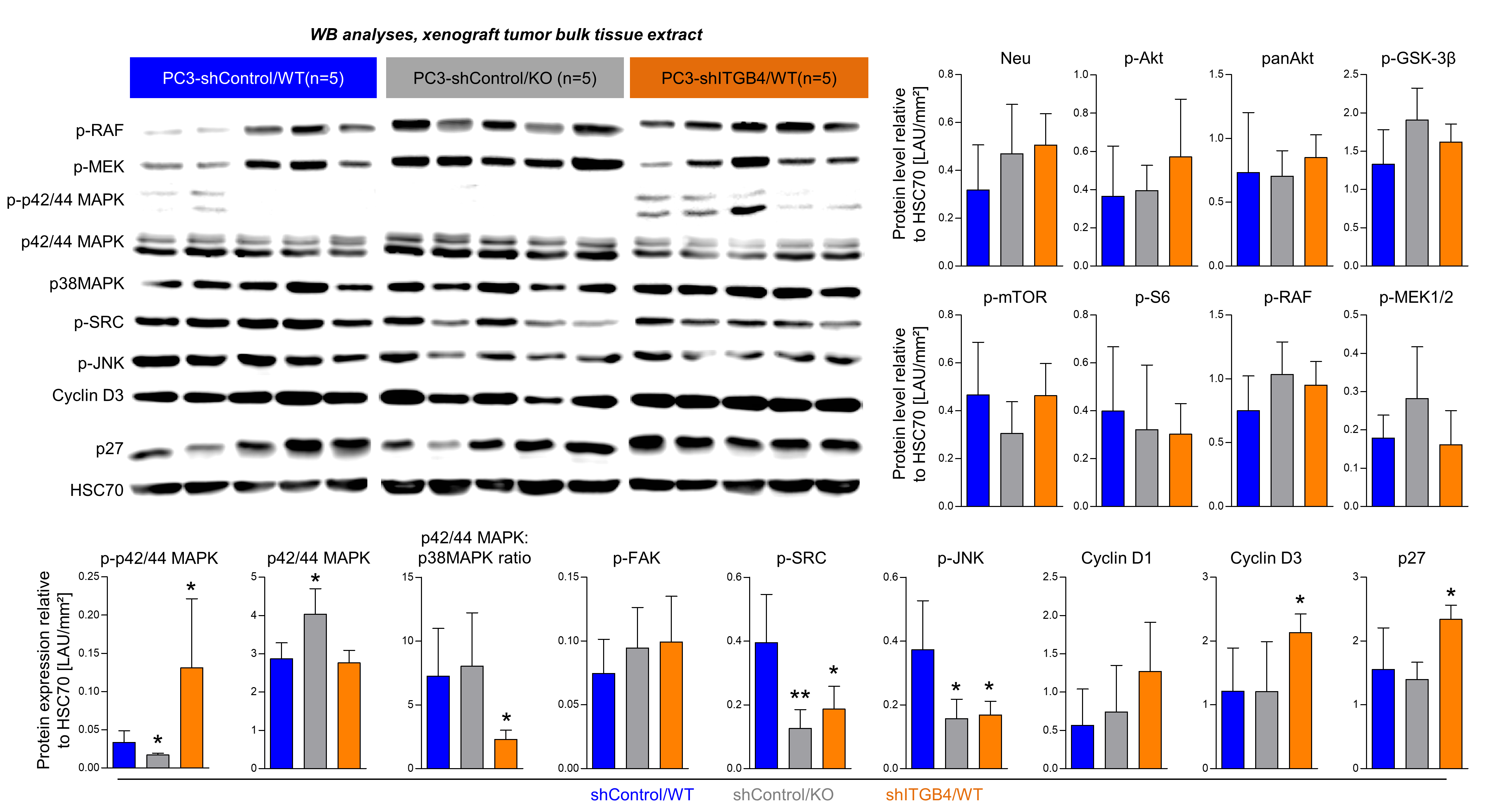

Supplement: Supplementary file 3 — Additional file 3. Suppl. Fig. S3: Western Blot analyses of PC-3 xenograft tumor samples. Exemplary Western Blot images and corresponding quantification of cell cycle- and survival-related protein levels relative to HSC70. Proteins were extracted from PC-3 xenograft tumors from the experiment shown in Fig. 1D. The combination group (shITGB4/KO) is missing since in this group only one mouse developed a xenograft tumor. Bar charts represent mean+SD of n=5. *p<0.05; **p<0.01. [file 13045_2023_1413_MOESM3_ESM.tif]

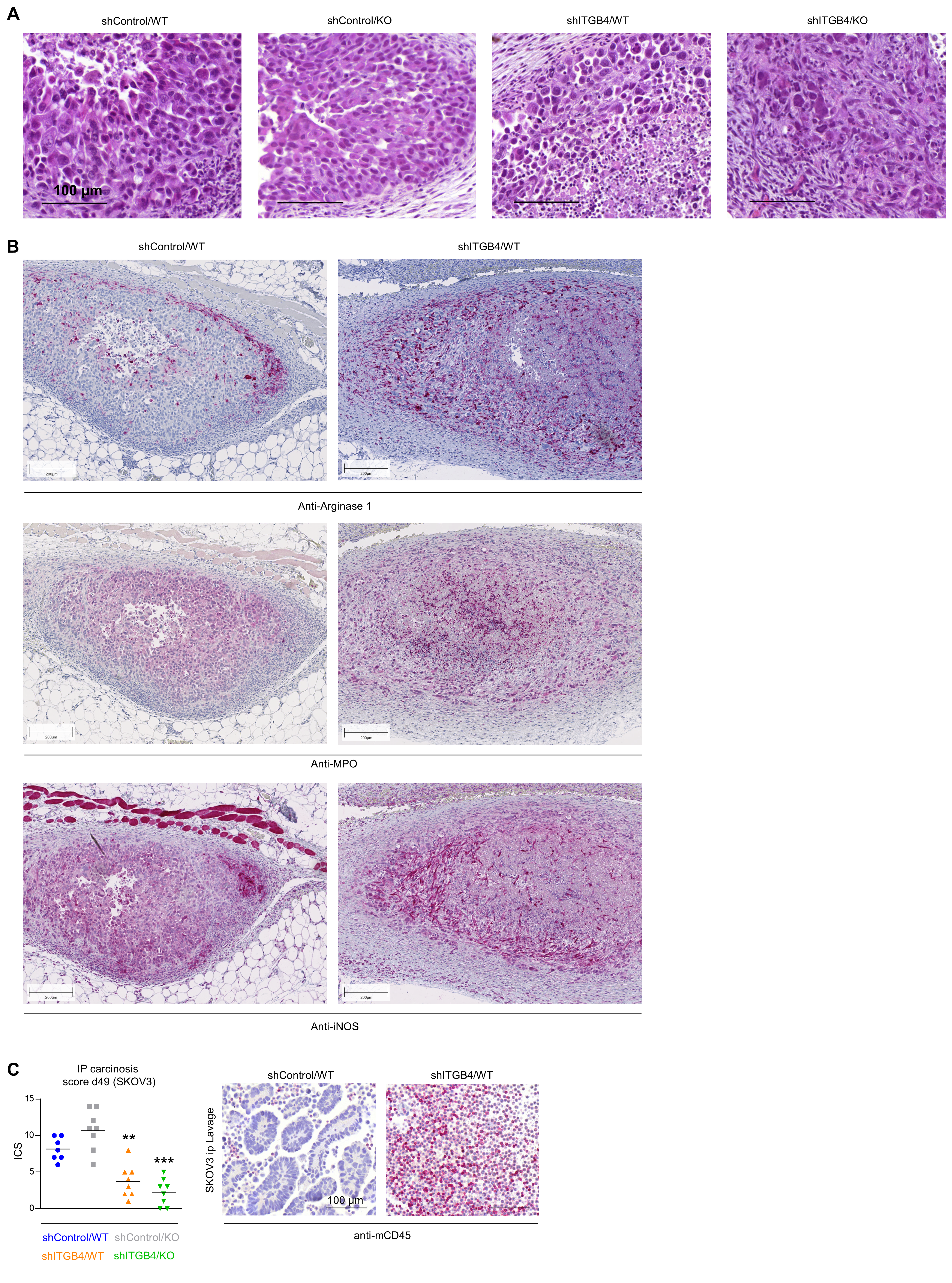

Supplement: Supplementary file 4 — Additional file 4. Suppl. Fig. S4: Enhanced attraction of tumor-infiltrating leukocytes in ITGB4-depleted xenografts. Morphology of control and ITGB4 KD tumors in WT and KO mice based on HE stainings (A). Arginase-1, myeloperoxidase (MPO) and inducible nitric oxide synthase (iNOS) expression in control vs. ITGB4 KD PC-3 tumor nodules on d8 after engraftment (B). Intraperitoneal (IP) carcinosis score (ICS) on day 49 after injection of SKOV3 control vs. ITGB4 knockdown cells into E-/P-selectin wildtype vs. knockout rag2-/- BALB/c mice. Representative anti-mCD45 immunostaining images of formalin-fixed, paraffin-embedded intraperitoneal lavage (C). Black lines in the scatter plot represent mean values. **p<0.01; ***p<0.001. [file 13045_2023_1413_MOESM4_ESM.tif]

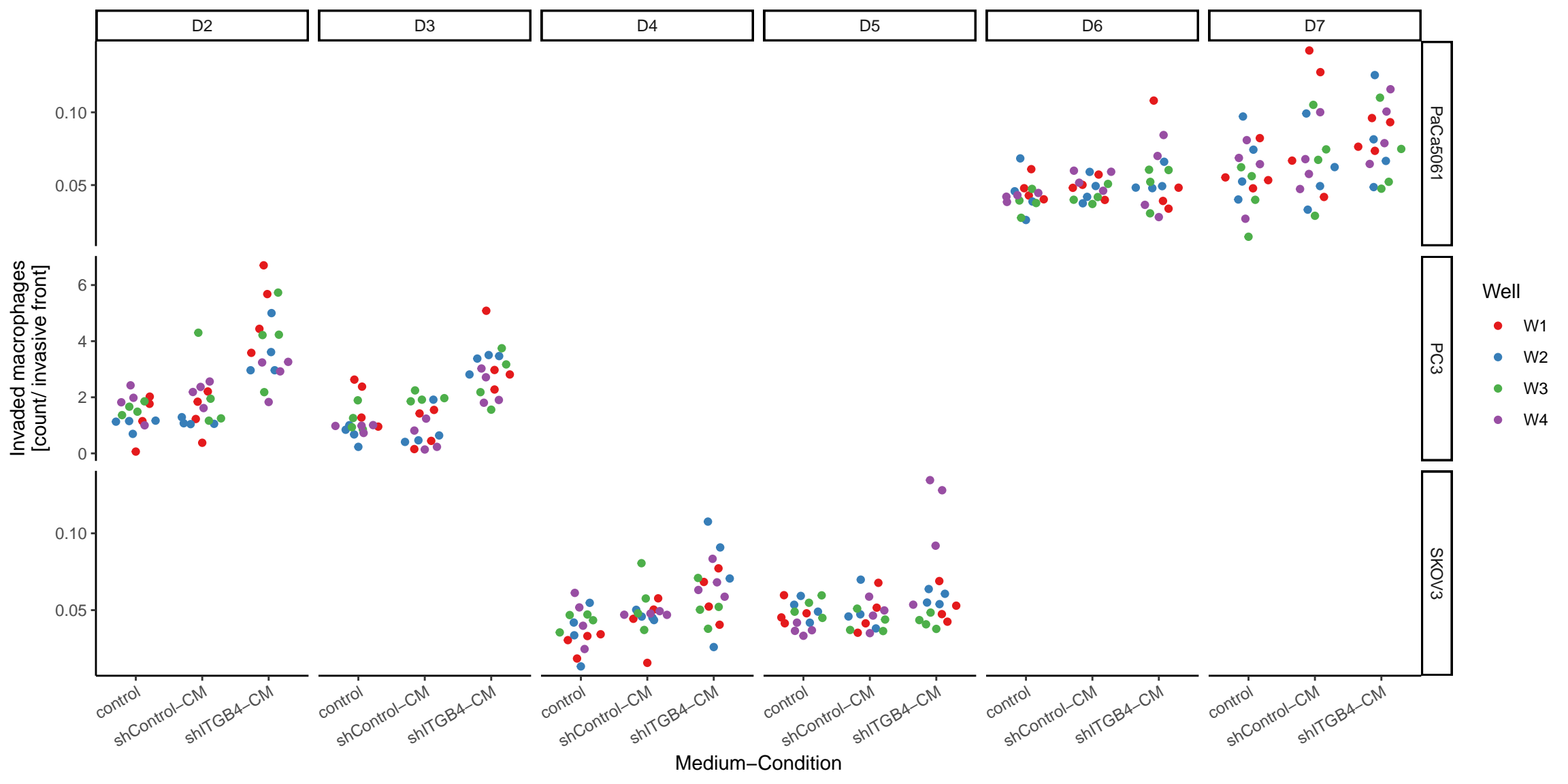

Supplement: Supplementary file 5 — Additional file 5. Suppl. Fig. S5: Attraction of human macrophages by ITGB4 knockdown tumor cell-conditioned media. Entire dataset of data shown in Fig. 7A including further donors. [file 13045_2023_1413_MOESM5_ESM.pdf]

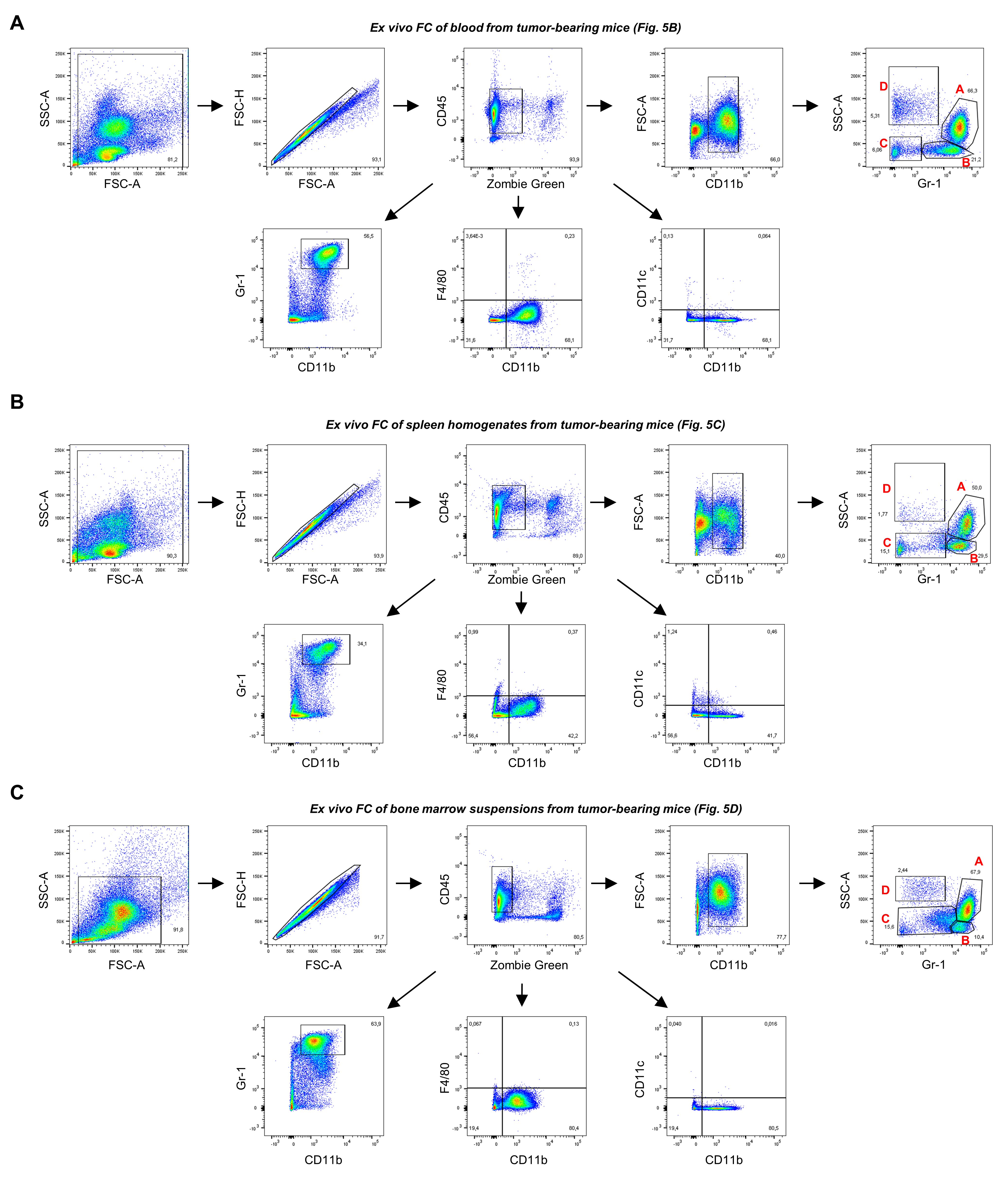

Supplement: Supplementary file 6 — Additional file 6 Suppl. Fig. S6: Representative density plots of ex vivo flow cytometric analyses of blood (A), spleen (B) and bone marrow (C) samples of s.c. tumor nodule-bearing mice (10 days after injection of PC-3 control vs. ITGB4 knockdown cells into E-/P-selectin wildtype vs. knockout mice, see Fig. 8 for differences between the groups). Note the populations assigned A-D in SSC-A/ Gr-1 plots (referring to Fig. 8). [file 13045_2023_1413_MOESM6_ESM.tif]

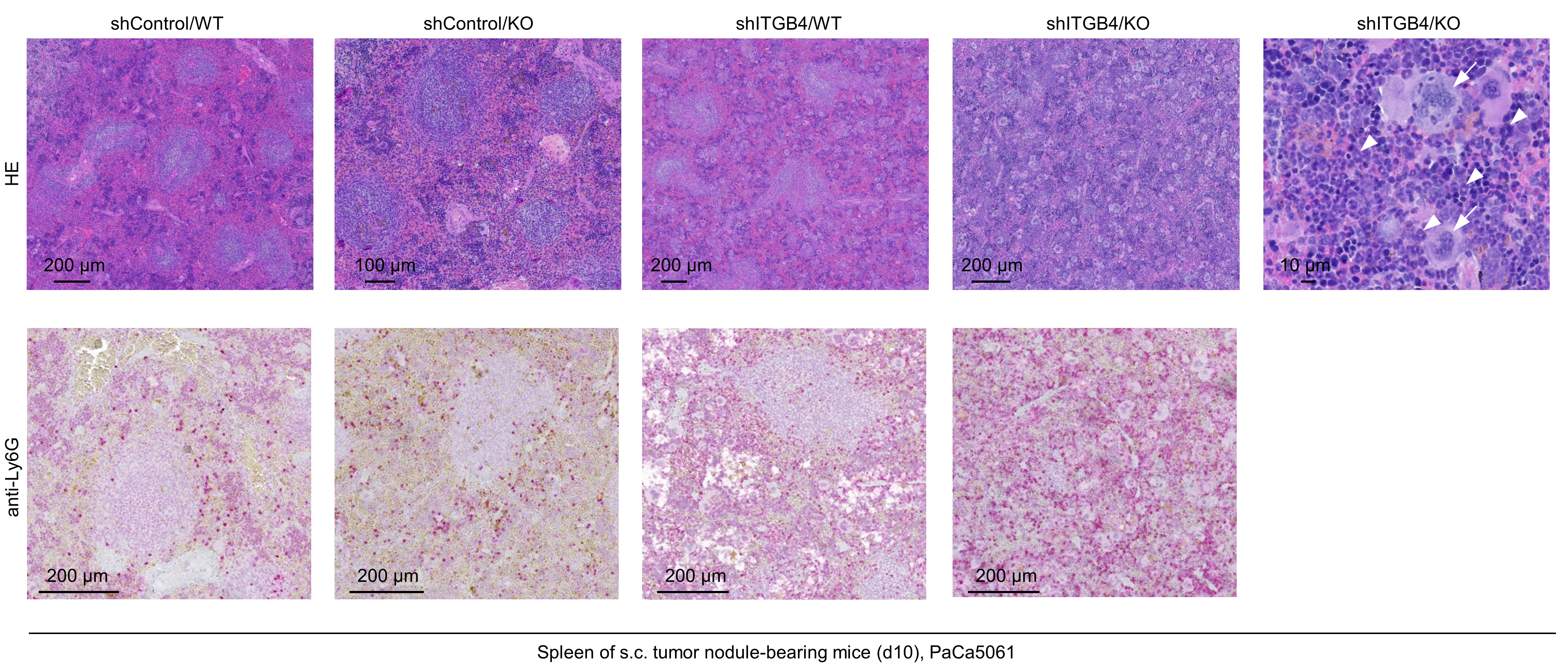

Supplement: Supplementary file 7 — Additional file 7. Suppl. Fig. S7: Validation of alterations in the spleen morphology ten days after s.c. injection of tumor cells. Representative photomicrographs of HE- and anti-Ly6G (Gr-1 epitope)-stained spleen samples on d10 after s. c. injection of PaCa5061 control vs. ITGB4 knockdown cells into E-/P-selectin wildtype vs. knockout mice. The arrows indicate megakaryocytes, arrowheads indicate immature granulocytes with ring-shaped nuclei. [file 13045_2023_1413_MOESM7_ESM.tif]

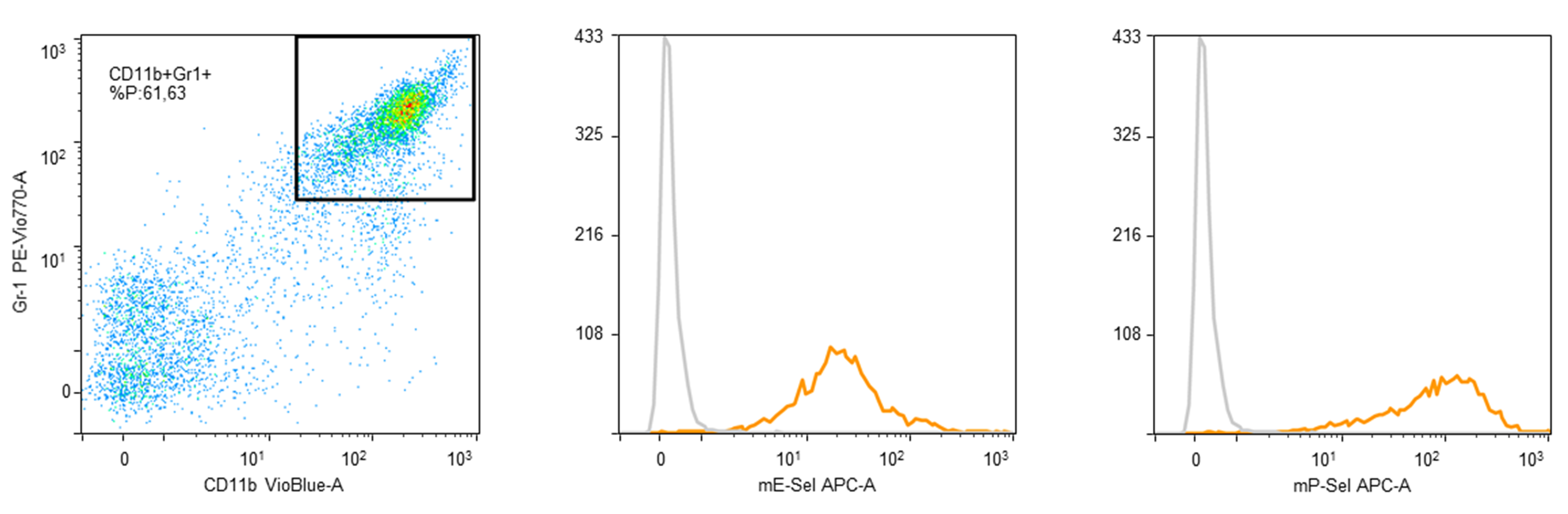

Supplement: Supplementary file 8 — Additional file 8. Suppl. Fig. S8: Static murine E- and P-selectin binding capacity of ITGB4 KD tumor-derived MDSCs. Flow cytometric analysis of CD11b+ Gr-1+ leukocytes isolated from s.c. PC-3 ITGB4 KD tumors regarding static binding of murine E- and Pselectin as indicated. Grey lines in histograms indicate unstained controls. [file 13045_2023_1413_MOESM8_ESM.tif]

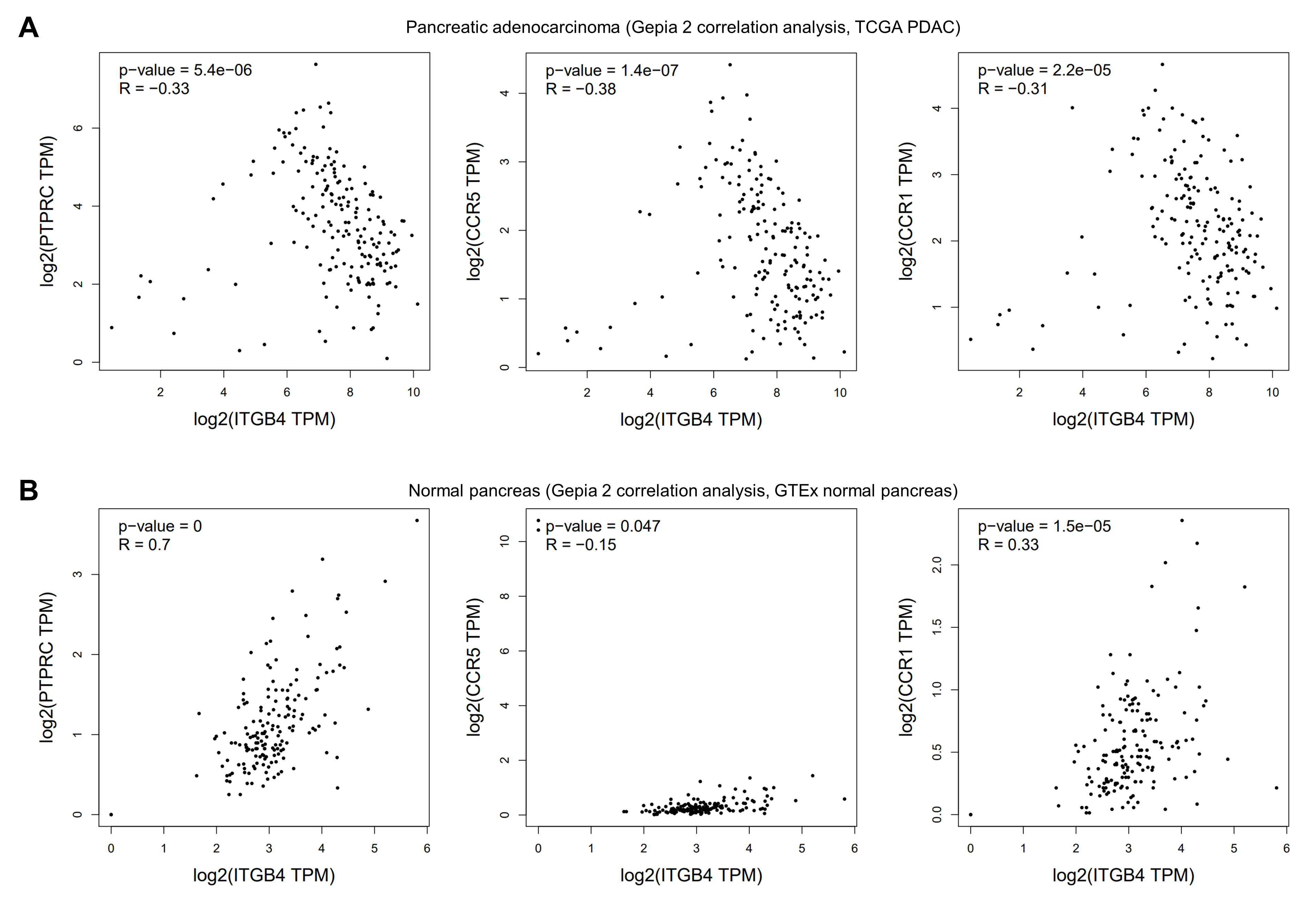

Supplement: Supplementary file 9 — Additional file 9. Suppl. Fig. S9: Validation of inverse correlation of ITGB4 and leukocyte markers in pancreatic cancer. Correlation of ITGB4 and CD45 (PTPRC gene), CCR5, or CCR1 in the human pancreatic ductal adenocarcinoma (PDAC) database of the cancer genome atlas (TCGA) (A) and normal human pancreas expression data of the Genotype-Tissue Expression (GTEx) portal (B) as determined by using the Gene Expression Profiling Interactive Analysis 2 (GEPIA2) online tool (http://gepia2.cancer-pku.cn). TPM = transcripts per million. R- and p-values were calculated based on Pearson correlation analysis. [file 13045_2023_1413_MOESM9_ESM.tif]
