# Supplementary material for: Tumor cell integrin β4 and tumor stroma E-/P-selectin cooperatively regulate tumor growth in vivo
Source: J Hematol Oncol. 2023 Mar 17;16:23. doi: 10.1186/s13045-023-01413-9 (PMC10022201; doi:10.1186/s13045-023-01413-9)
Supplement: Supplementary file 10 — Additional file 10. Suppl. Table 1: Short IHC protocols relevant to the study (IHC-P). [file 13045_2023_1413_MOESM10_ESM.docx]

**Suppl. Table 1:** Short IHC protocols relevant to the study (IHC-P).

|  | Antigen retrieval | primary antibody | isotype control | secondary antibody |
| --- | --- | --- | --- | --- |
| *Integrin beta 4 (439-9B)* | S1699 pH6,  10 min,  121°C steamer | abcam  ab110167 (final conc.:  20 µg/ml) | rat IgG2b Biolegend | rabbit α rat (Jackson ImmunoResearch) 1:100 |
| *Ki67 (M7240)* | S1699 pH6,  16h  85°C water bath | Dako  M7240  (1.1 µg/ml) | mouse IgG1  Invitrogen | goat α mouse (LS-Bio) 1:200 |
| *Bim* | S2367 pH9, (Tris/EDTA)  10 min  121°C steamer | abcam ab137416  (10 µg/ml) | rabbit poly IgG abcam | swine α rabbit (Dako) 1:200 |
| *pH2A.X [EP854(2)Y]* | S1699 pH6,  10 min  121°C steamer | abcam  ab81299 (final conc.: 0.711 µg/mL) | rabbit Ig fraction Dako | goat α rabbit (LS-Bio) 1:200 |
| *mCD45 (30-F11)* | S1699 pH6,  2 x 4 min microwave | BD Pharmingen 550539  (final conc.: 2.5 µg/mL) | rat IgG2b BioLegend | rabbit α rat  (Jackson Immono Research) 1:100 |
| *Ly6G* | S2367 pH9, (Tris/EDTA)  10 min,  121°C steamer | MyBioSource MBS2556115  (final conc.: 3.6 µg/mL) | rabbit poly IgG abcam | goat α rabbit (LS-Bio) 1:200 |
| *mIntegrin beta 4 [EPR17517]* | S2367 pH9 (Tris/EDTA) | abcam  ab18212  (final conc.: 5 µg/mL) | rabbit mono IgG abcam | goat α rabbit (LS-Bio) 1:200 |
| *mCD3* | S1699 pH6,  10 min,  121°C steamer | abcam ab16669  (final conc.: ca. 4 µg/mL) | rabbit mono IgG abcam | goat α rabbit (LS-Bio) 1:200 |
| *mCD8* | S1699 pH6,  2 x 5 min microwave | Cell Signaling #98941  Conc? | rabbit poly IgG abcam | goat α rabbit (LS-Bio) 1:200 |
| *mArginase 1*  *[EPR22033-369]* | S2367 pH9,  (Tris/EDTA)  20 min  100°C steamer | abcam  ab 233548  (final conc.: 1 µg/mL) | rabbit mono IgG abcam | goat α rabbit (LS-Bio) 1:200 |
| *mMyeloperoxidase* | S1699 pH6,  20 min  100°C steamer | abcam  ab139748  (final conc.: 5 µg/mL) | rabbit poly IgG abcam | goat α rabbit (LS-Bio) 1:200 |
| *miNOS* | S1699 pH6,  10 min  121°C steamer | GeneTex  GTX130246  (final conc.: 3 µg/mL) | rabbit poly IgG abcam | goat α rabbit (LS-Bio) 1:200 |
